# Population genomics of *Plasmodium ovale* species in sub-Saharan Africa

**DOI:** 10.1038/s41467-024-54667-3

**Published:** 2024-11-27

**Authors:** Kelly Carey-Ewend, Zachary R. Popkin-Hall, Alfred Simkin, Meredith Muller, Chris Hennelly, Wenqiao He, Kara A. Moser, Claudia Gaither, Karamoko Niaré, Farhang Aghakanian, Sindew Feleke, Bokretsion G. Brhane, Fernandine Phanzu, Melchior Mwandagalirwa Kashamuka, Ozkan Aydemir, Colin J. Sutherland, Deus S. Ishengoma, Innocent M. Ali, Billy Ngasala, Albert Kalonji, Antoinette Tshefu, Jonathan B. Parr, Jeffrey A. Bailey, Jonathan J. Juliano, Jessica T. Lin

**Affiliations:** 1 Department of Epidemiology, Gillings School of Global Public Health, University of North Carolina, Chapel Hill, NC USA; 2 Institute for Global Health and Infectious Diseases, University of North Carolina, Chapel Hill, NC USA; 3 Department of Pathology and Laboratory Medicine, Brown University, Providence, RI USA; 4 Ethiopian Public Health Institute, Addis Ababa, Ethiopia; 5 SANRU Asbl, Kinshasa, Democratic Republic of the Congo; 6 Kinshasa School of Public Health, Kinshasa, Democratic Republic of the Congo; 7 Program in Molecular Medicine, Chan Medical School, University of Massachusetts, Worcester, MA USA; 8 London School of Hygiene and Tropical Medicine, London, UK; 9 National Institute for Medical Research (NIMR), Dar es Salaam, Tanzania; 10 Department of Biochemistry, Kampala International University in Tanzania, Dar es Salaam, Tanzania; 11 Department of Biochemistry, Faculty of Science, University of Dschang, Dschang, Cameroon; 12 Muhimbili University of Health and Allied Sciences, Dar es Salaam, Tanzania; 13 Division of Infectious Diseases, University of North Carolina School of Medicine, University of North Carolina, Chapel Hill, NC USA; 14 Curriculum in Genetics and Molecular Biology, University of North Carolina School of Medicine, University of North Carolina, Chapel Hill, NC USA; 15 Department of Microbiology and Immunology, University of North Carolina School of Medicine, University of North Carolina, Chapel Hill, NC USA

**Keywords:** Malaria, Population genetics

## Abstract

*Plasmodium ovale curtisi* (*Poc)* and *Plasmodium ovale wallikeri* (*Pow*) are relapsing malaria parasites endemic to Africa and Asia that were previously thought to represent a single species. Amid increasing detection of ovale malaria in sub-Saharan Africa, we present a population genomic study of both species across the continent. We conducted whole-genome sequencing of 25 isolates from Central and East Africa and analyzed them alongside 20 previously published African genomes. Isolates are predominantly monoclonal (43/45), with their genetic similarity aligning with geography. *Pow* shows lower average nucleotide diversity (1.8×10^−4^) across the genome compared to *Poc* (3.0×10^−4^) (p < 0.0001). Signatures of selective sweeps involving the dihydrofolate reductase gene have been found in both species, as are signs of balancing selection at the merozoite surface protein 1 gene. Differences in the nucleotide diversity of *Poc* and *Pow* may reflect unique demographic history, even as similar selective forces facilitate their resilience to malaria control interventions.

## Abstract (web summary)

Genomic analyses, performed by Carey-Ewend et al., reveal that Plasmodium ovale curtisi and wallikeri in sub-Saharan Africa show similar low complexity of infection, relatedness by geography, and signatures of selection. However, P. ovale wallikeri harbors lower nucleotide diversity.

## Introduction

Parasites in the genus *Plasmodium* were responsible for an estimated 249 million cases of malaria and 608,000 deaths in 2022^1^. Ninety-four percent of these cases occurred in the World Health Organization Africa Region, where control efforts have primarily focused on the predominant species, *P. falciparum* (*Pf*)^2^. Yet these case counts likely underrepresent the burden of non-falciparum species, which may be rising in prevalence even where control efforts have successfully reduced *P. falciparum* transmission^3–5^. Over the last few decades, genomic studies of *P. falciparum* have enabled monitoring of drug resistance markers^6^, facilitated the identification of promising vaccine candidates^7^, uncovered the structure of parasite populations^8^, and identified evolutionary forces shaping their demography^9,10^. Much less is known about non-falciparum species, especially their comparative evolutionary history and susceptibility to malaria control interventions focused on *P. falciparum*.

*Plasmodium ovale* was first identified as a separate malaria species in 1922 based on the appearance of oval-shaped erythrocytes that contained non-ring parasite forms^11^. Hallmarks of this parasite species are its restriction to younger red cells and, therefore, the propensity to cause low-density infections, as well as relapses from liver hypnozoites, similar to *P. vivax* and *P. cynomologi*. The species often causes coinfection alongside *P. falciparum* which, along with its low parasite densities, makes it challenging to differentiate morphologically on peripheral blood smears^12^. The advent of polymerase chain reaction (PCR)-based diagnostics has improved the detection of ovale infections, but initial PCR surveys across Africa and Asia based on the small subunit rRNA gene revealed two apparent groups of *P. ovale* parasites, termed classic and variant^13^. The discovery of perfect sequence segregation of six genomic markers, and more recently, 12 mitochondrial loci, between classic and variant *P. ovale* isolates collected across Africa and Asia has led to the conclusion that this dimorphism actually represents a true species divide in the *P. ovale* clade^14,15^. The nomenclature of these species is currently evolving but will be referred to as *P. ovale curtisi* (*Poc*, formerly classic) and *P. ovale wallikeri* (*Pow*, formerly variant) herein^16–18^.

*P. ovale curtisi and P. ovale wallikeri* have since been confirmed to circulate within the same human populations throughout Africa and Asia^19,20^, with both detected by PCR at higher rates than previously appreciated^5,12,21,22^. Limited investigation of the genetic diversity and population genetics of the two *P. ovale* species have hinted at low diversity and/or small effective population size, as few unique haplotypes have been identified at antigenic gene targets like apical membrane antigen 1 (*ama1*) and merozoite surface protein 1 (*msp1*)^20,23^. There is some indication that drugs used to treat *P. falciparum* are also shaping *P. ovale* parasite populations; signs of a selective sweep involving a mutant *dhfr* allele (implicated in pyrimethamine resistance) have been detected in both *Poc* and *Pow*^24,25^. Until now, the low density of most *P. ovale* isolates combined with the lack of an in vitro culture system has hindered whole-genome sequencing of these parasites^11^. However, with the development of strategies for parasite DNA enrichment, as well as the construction of the first reference genomes in 2017, genome-wide analyses are now possible^15,26–28^.

In this work, we employ hybrid capture or leukodepletion to enrich *P. ovale* spp. DNA and perform whole-genome sequencing (WGS) of 25 clinical isolates collected from studies conducted across Ethiopia, the Democratic Republic of the Congo, Tanzania, and Cameroon. Combined with 20 additional public whole genomes from 11 countries spanning East, Central, and West Africa, we seek to better understand the comparative biology of *P. ovale curtisi*, *P. ovale wallikeri*, and co-endemic *P. falciparum* by examining their complexity of infection, population structure, nucleotide diversity, and genomic signatures of selection.

## Results

### High-quality genomic coverage of African *P. ovale* isolates

Parasite samples from 25 *P. ovale*-infected individuals collected at ten sites spanning Ethiopia, the Democratic Republic of the Congo (DRC), Tanzania, and Cameroon were selected for whole-genome sequencing (Table 1)^29–34^. These included 13 *P. ovale curtisi* and 12 *P. ovale wallikeri* isolates that were selected from six studies based on robust amplification of the *po18S* rRNA gene (Ct < 36) and predominance of one *ovale* species within each isolate. The majority of the samples (*n* = 21) underwent a custom-designed hybrid capture with RNA baits to preferentially isolate ovale DNA extracted from dried blood spots for sequencing, while four additional whole blood samples were leukodepleted (LDB) at the time of collection by CF11 filtration and directly sequenced without enrichment^35^. Finally, genomic data of 20 *P. ovale* isolates sequenced as part of four previously published studies were retrieved from the European Nucleotide Archive and the Sequence Read Archive^15,28,36,37^. These isolates either underwent selective whole-genome amplification (sWGA) or leukodepletion for parasite DNA enrichment prior to sequencing. Further data on all parasite isolates are found in Supplemental Table 1.

**Table 1:** Studies of origin for 45 *P*. *ovale* isolates

Study	Country of origin	Year of collection	Study population	# selected for sequencing (# of *Poc* and *Pow*)
*hrp*2/3 Deletion Survey^29^	Ethiopia	2017– 2018	Febrile patients presenting to health facilities in the Amhara, Tigray, and Gambella regions	7 (2 *Poc*, 5 *Pow*)
SANRU Rural Health Program^30^	Democratic Republic of the Congo	2017	Febrile patients presenting to health facilities in Sud-Kivu, Bas-Uele, and Kinshasa Provinces	6 (2 *Poc*, 4 *Pow*)
Kinshasa Malaria Longitudinal Study^31^	Democratic Republic of the Congo	2015– 2017	Members of households participating in longitudinal study of malaria	5 (5 *Poc*)
TranSMIT^32^	Tanzania (East)	2018– 2022	Asymptomatic children and adults attending school or health clinics in rural Bagamoyo district, eastern Tanzania	4 (3 *Poc*, 1 *Pow*)
MSMT21^33^	Tanzania (West)	2020– 2022	Tanzanian citizens at health facilities	2 (2 *Pow*)
Dschang Febrile Cohort^34^	Cameroon	2020–2021	Febrile patients presenting to health facilities in western Cameroon	1 (1 *Poc*)
Joste et al., *JID* 2023^36^ *	Cameroon, Senegal, Ivory Coast	2013–2021	*P. ovale* infections identified in France after travel to an endemic country	4 (4 *Pow*)
Rutledge et al., *Nature* 2017^28^ *	Ghana, Cameroon	Unknown	Symptomatic *P. falciparum* infected individuals with strong *P. ovale* signals among whole-genome sequencing results	2 (1 *Poc*, 1 *Pow*)
Higgins et al., *Sci Rep*. 2024^15^ *	Tanzania, Kenya, South Sudan, Congo, Cameroon, Nigeria, Sierra Leone	2019– 2020	*P. ovale* infections identified in the United Kingdom after travel to an endemic country.	11 (6 *Poc*, 5 *Pow*)
Ansari et al., *Int J Parasitol*. 2016^37^ *	Gabon, Nigeria	Unknown	Febrile Chinese males presenting to clinics in Jiangsu Province, China, after travel to an endemic country	3 (1 *Poc*, 2 *Pow*)

*Raw sequencing data for these 20 isolates were directly incorporated into the analysis pipeline after retrieval from the European Nucleotide Archive or NCBI Sequence Read Archive.

**Fig. 1:**
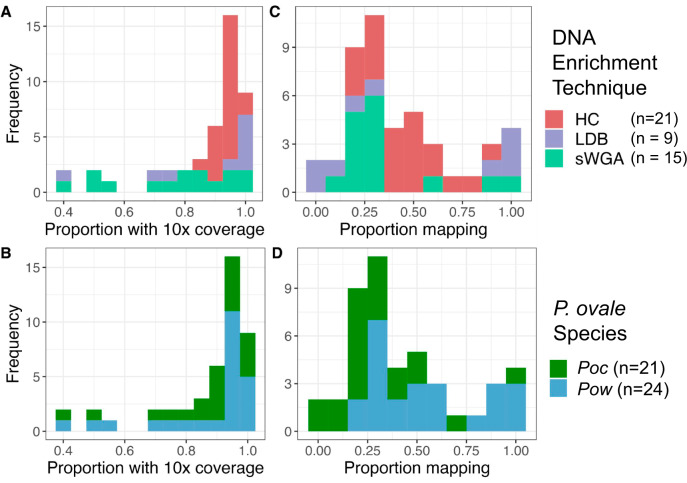
Coverage and mapping among 45 isolates. The proportion of the corresponding *P. ovale* reference genome covered by ≥10 reads by DNA enrichment technique (**A**) and species (**B**). The proportion of reads mapped to that reference genome by DNA enrichment technique (**C**) and species (**D**). For *Poc*, chromosome 10 was excluded due to incomplete coverage by hybrid capture baits. Five samples from Higgins et al. were not incorporated into this study due to having <30% 10x coverage of the corresponding genome. HC hybrid capture, LDB leukodepleted blood, sWGA selective whole-genome amplification. Source data are provided as a Source Data file.

Whole-genome sequencing achieved high genome coverage, with an overall average of 86 and 87% tenfold coverage across the core genome for the 21 *P. ovale curtisi* and 24 *P. ovale wallikeri* isolates, respectively (Fig. 1). Coverage and mapping proportion were highest when aligned to the *P. ovale* reference genome determined by the *Poc*/*Pow* species-specific qPCR assay, corroborating initial species assignment. Compared to sWGA and LDB samples, the hybrid capture method used to enrich parasite DNA in the majority of samples yielded more complete coverage across all chromosomes except for chromosome 10 (Fig. 1). The hybrid capture was originally designed for *P. ovale wallikeri*, with additional *P. ovale curtisi* baits then selected to cover areas that differ between the two ovale genome assemblies (PowCR01 and PocGH01)^28^. Due to *Pow* chromosome 10 being incomplete in the PowCR01 reference genome (only 470 kb), this approach did not provide coverage for the full *Poc* chromosome 10 (1300 kb). This led to substantially lower coverage for chromosome 10 across all *Poc* hybrid capture isolates (40–60% 10x coverage vs. >85% for all other *Poc* chromosomes); thus, chromosome 10 was excluded from all genome-wide analysis in *Poc* isolates to limit error.

As expected, hybrid capture led to preferential sequencing of *P. ovale* DNA among samples that were co-infected with *P. falciparum* (*Pf*); *Pf*-positive isolates that underwent hybrid capture yielded only 2–11% 10x coverage of the *Pf* genome compared to >90% 10x coverage of the *Pf* genome among leukodepleted blood samples. For genomic analysis, insertions/deletions, multiallelic sites, low-quality variants, and variants within tandem repeats and expanded gene families were excluded (see Methods), yielding final biallelic single nucleotide polymorphism (SNP) call sets of 73,015 SNPs for *P. ovale curtisi* and 45,669 for *P. ovale wallikeri*.

### Low complexity of infection

**Fig. 2:**
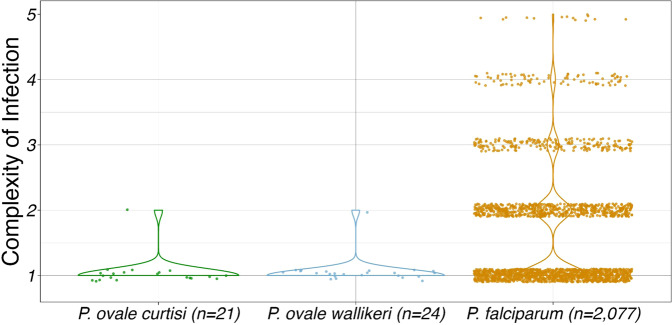
Complexity of infection by *Plasmodium* species. Median estimated complexity of infection (COI) among 21 *Poc* isolates, 24 *Pow* isolates, and 2077 *P. falciparum* isolates geographically matched to the *P. ovale* samples by country of origin. The distributions of COI differed significantly among the three species (*p* < 0.0001) by a Kruskal–Wallis test, with *Pow* and *Poc* showing significantly lower COI than *P. falciparum* (*p* = 0.0004 and 0.012, respectively) in Dunn’s multiple comparisons tests. The average read depth of coverage for *Poc*, *Pow*, and *Pf* isolates were 44.1, 95.7, and 147.5, respectively. Source data are provided as a Source Data file.

Complexity of infection (COI), or the number of unique parasite clones present in a given isolate, was estimated 1000 times using THEREALMcCOIL for all 21 *P. ovale curtisi* and 24 *ovale wallikeri* isolates, as well as 2077 geographically matched *P. falciparum* isolates downloaded from the publicly available MalariaGEN Pf6 dataset (Fig. 2)^38^. Twenty out of 21 *Poc* isolates (95%) and 23 out of 24 *Pow* isolates (96%) were estimated to be monoclonal; the remaining isolate in each *P. ovale* species was found to comprise two parasite clones. By comparison, roughly half (1165/2077; 56%) of *P. falciparum* samples were monoclonal. COI differed significantly (*p* = 0.005) among the three *Plasmodium* species. In pairwise comparisons, both *Poc and Pow* had significantly lower COI compared to *P. falciparum* (*p* = 0.001 and *p* = 0.004, respectively).

The two multiclonal *P. ovale* infections were both hybrid capture-enriched, high coverage (94 and 98% tenfold coverage in *Poc* and *Pow*, respectively) and came from high-transmission areas of the DRC^39^; each had a COI of 2. In order to determine whether the two clones in these samples were distinct lineages or meiotic siblings, we analyzed the distribution of heterozygous SNPs across the genome. We hypothesized that meiotic siblings would only have heterozygous SNPs in specific regions, reflecting recombination within the mosquito midgut^40^. In both samples, after filtering to high-confidence SNPs based on population-wide allele frequency, we saw an even distribution of heterozygous SNPs across the genome, suggestive of two distinct parasite lineages in the same host rather than meiotic siblings (Supplemental Fig. 1).

### Lower nucleotide diversity in *P. ovale wallikeri* compared to *P. ovale curtisi*

**Fig. 3:**
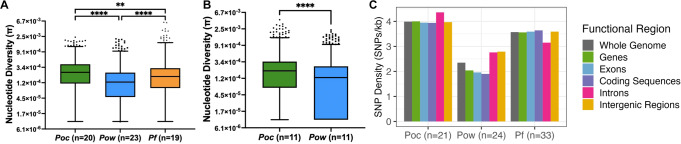
Nucleotide diversity (π) of orthologous genes and SNP density by functional genomic region among *Poc*, *Pow*, and *P. falciparum* (*Pf*) isolates. **A** Nucleotide diversity (**π**) per gene among 2008 sets of orthologous genes in monoclonal *Poc*, *Pow*, and *Pf* samples. Boxes denote the 25th, median, and 75th percentiles; whiskers are drawn at the 1st and 99th percentiles. **π** of 0 was coded as 1 × 10^−5^ to plot on a logarithmic scale. Nucleotide diversity was significantly different between orthologues of all three species by two-sided Tukey’s multiple comparisons tests, with *Poc* orthologues showing higher diversity than orthologues of *Pow* and *P. falciparum*, and *Pow* orthologues also showing lower diversity than those in *P. falciparum* (*p* values <0.001, =0.002, and <0.0001, respectively). **B** Nucleotide diversity (**π**) per gene among 2911 sets of orthologous genes in geographically matched monoclonal *Poc* and *Pow* samples. Boxes denote the 25th, median, and 75th percentiles; whiskers are drawn at the 1st and 99th percentiles. **π** of 0 was coded as 1 × 10^−5^ to plot on a logarithmic scale. Nucleotide diversity was significantly lower among *Pow* orthologues compared to *Poc* using a two-sided Wilcoxon’s matched-pair signed rank test (*p* < 0.0001). **C** SNP density in different functional regions of the genome among all *Pow*, *Poc*, and *P. falciparum* isolates. SNP single nucleotide polymorphism, kb kilobase. Source data are provided as a Source Data file.

Among a collection of 3339 sets of one-to-one orthologous genes between the *Poc*, *Pow*, and *P. falciparum* genomes, we identified 2008 sets that achieved high-quality sequencing coverage and had no overlap with masked genomic regions in any of the three species. The average species-specific nucleotide diversity (**π**) among these orthologues in the 20 monoclonal *Poc*, 23 monoclonal *Pow*, and 19 geographically matched monoclonal *Pf* samples were 2.9 × 10^−4^, 1.8 × 10^−4^, and 2.6 × 10^−4^, respectively. These were significantly different between species (*p* < 0.0001, *F* = 98, df = 2), with orthologues in *P. ovale curtisi* more diverse than in *P. ovale wallikeri* and *P. falciparum* (*p* values <0.0001 and 0.002, respectively), and *Pow* orthologues less diverse than in *Pf* (*p* < 0.0001) (Fig. 3A). To mitigate bias by geographic coverage and orthology with the *P. falciparum* genome, we repeated this analysis using 2,911 *Poc*-*Pow* orthologues among a group of geographically matched monoclonal *Poc* and *Pow* samples (*n* = 11 each, Supplemental Table 3), revealing average nucleotide diversities of 2.5 × 10^−4^ and 1.8 × 10^−4^, respectively (Fig. 3B). Nucleotide diversity was still significantly lower in *Pow* orthologues compared to *Poc* (*p* < 0.0001).

This high nucleotide diversity in *P. ovale curtisi* was consistent with an investigation of the total number and density of genome-wide SNPs. Variant calling and filtering resulted in almost twice the number of SNPs for *P. ovale curtisi* (73,015) as for *P. ovale wallikeri* (45,669), despite a slightly smaller number of *Poc* isolates (21 vs. 24). This corresponded to a higher density of SNPs across the *Poc* genome (4.0 SNPs per kilobase[kb] in *Poc* vs. 2.4 SNPs/kb in *Pow*). However, a smaller proportion of SNPs in the *Poc* genome were nonsynonymous mutations, as the ratio of nonsynonymous-to-synonymous (dN/dS) mutations was 1.5 and 2.5 in *Poc* and *Pow*, respectively. Among the aforementioned geographically matched *P. ovale* isolates, dN/dS within 2911 orthologous genes was 1.4 for *Poc* and 2.5 for *Pow*, consistent with the broader estimates. SNP densities in both the *Poc* and *Pow* genomes were lowest in protein-coding sequences (3.9 and 1.9 SNPs/kb, respectively), and higher in introns (4.4 and 2.8 SNPs/kb) (Fig. 3C). Intergenic regions in *Poc* showed relatively lower SNP density similar to protein-coding sequences (4.0 SNPs/kb), but these same regions in *Pow* had relatively high SNP density similar to introns (2.8 SNPs/kb).

### Parasite genomic similarity recapitulates geographic relationships

**Fig. 4:**
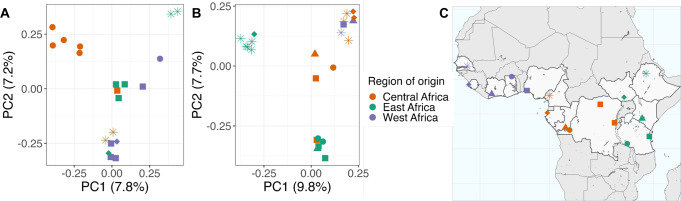
Principal component analysis of monoclonal *P. ovale* spp. isolates. Principal component analysis showing the first two principal components among 20 monoclonal *Poc* isolates (**A**) and 23 monoclonal *Pow* isolates (**B**) using 4116 and 3189 biallelic SNPs, respectively. Samples are colored by region of country of origin; in the map, parasites from travelers are assigned to the capital city (**C**). In PC2 of *Pow*, SNPs within both the *ts-dhfr* and *mrp1* gene were among the top 0.5% of contributors. Source data are provided as a Source Data file.

Genome-wide principal component (PC) analysis of the monoclonal samples of each ovale species revealed the spatial arrangement of related parasites along PC1 and PC2 that aligns with their location of origin (Fig. 4). These components accounted for 15.0 and 17.5% of the genetic differentiation in *Poc* and *Pow*, respectively. While cluster analysis by ADMIXTURE found the best fit when modeling each isolate as a separate cluster, except for one pair of isolates per species originating from the capital of Kinshasa in the DRC (in *Poc*) and the Amhara region in Ethiopia (in *Pow*), geographic alignment was evident in the PCA. For *Pow*, PC1 and PC2 appear to reflect an East-West axis and North-South axis, respectively, with samples from Ethiopia and South Sudan in the west divided from others by PC1. In the PCA for *Poc*, Ethiopian parasites were also organized separately from other samples, as did isolates from Kinshasa in the DRC. The remaining *Poc* samples show some division between East, Central, and West Africa, though the alignment with geography is less consistent than in *Pow*. In both *P. ovale* spp., PC3 and PC4 further separated samples from various countries (Supplemental Fig. 2).

Examination of the top 0.5% of variants by contribution to each of the first 4 principal components revealed that SNPs within genes encoding multidrug resistance protein 1 (*mdr1*) and dihydrofolate reductase-thymidylate synthase (*dhfr-ts*), two putative antimalarial resistance genes, were major contributors to the North-South axis in *Pow* PC2. Among all 24 *Pow* samples, three previously documented haplotypes in *Pow dhfr-ts*, a key gene in folate metabolism that is implicated in pyrimethamine resistance^41,42^, appear to drive this geographic differentiation, with the Phe57Leu + Ser58Arg haplotype existing in 45% of our Central African clones and 36% of our East African clones but none of the sequenced West African clones (Supplemental Fig. 3A)^25^. This haplotype is associated with resistance to pyrimethamine when expressed in *E. coli*. Though it did not drive differentiation in the PCA, *Poc dhfr-ts* haplotypes similarly showed the presence of a putative drug resistance haplotype (Ala15Ser + Ser58Arg) in the Central and East African clones but not in West Africa, though our sample size for West Africa was generally smaller for both species (three and six isolates in *Pow* and *Poc*, respectively) (Supplemental Fig. 3B).

### Signatures of selection contain putative drug resistance loci, proteins involved in sexual stage differentiation, and antigenic targets

**Fig. 5:**
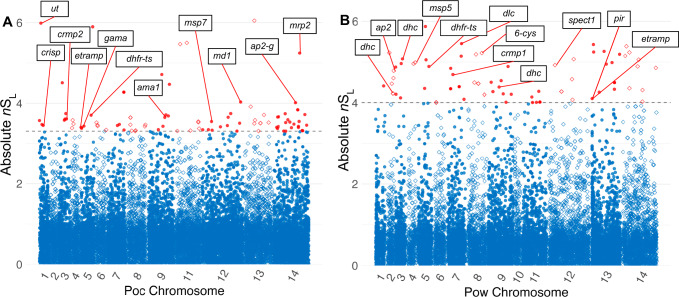
Absolute *n*S_L_ across the *Poc* and *Pow* genome. Absolute *n*S_L_ of 19,205 variants among monoclonal isolates across the *Poc* genome (**A**) and 15,744 variants among monoclonal isolates across the *Pow* genome (**B**). Individual loci are depicted using alternating shapes between chromosomes for legibility. The dotted line and red color denote the top 0.5% of loci. Putative genetic markers of note within 10,000 bases of these loci are labeled, including ubiquitin transferase (*ut*), cysteine-rich secretory protein (*crisp*), cysteine repeat modular protein 2 (*crmp2*), early transcribed membrane protein (*etramp*), GPI-anchored micronemal antigen (*gama*), dihydrofolate reductase-thymidylate synthase (*dhfr-ts*), apical membrane antigen (*ama1*), merozoite surface protein 7-like protein (*msp7*), male development protein 1 (*md1*), AP2 domain transcription factor G (*ap2-g*), multidrug resistance-associated protein 2 (*mrp2*), dynein heavy chain (*dhc*), AP2 domain transcription factors (*ap2*), merozoite surface protein 5 (*msp5*), cysteine repeat modular protein 1 (*crmp1*), dynein light chain (*dlc*), 6-cysteine protein (*6-cys*), sporozoite protein essential for cell traversal 1 (*spect1*), *Plasmodium* interspersed repeat protein (*pir*), and early transcribed membrane protein (*etramp*). Source data are provided as a Source Data file.

**Fig. 6:**
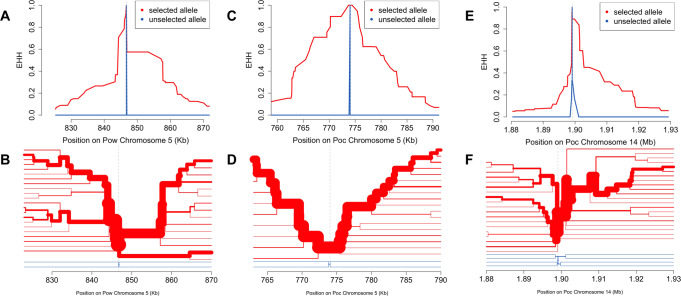
Extended haplotype homozygosity and haplotype bifurcation at selected variants. Extended haplotype homozygosity (EHH) and haplotype bifurcation among monoclonal isolates at selected variants near the *Pow dhfr-ts* gene (**A**, **B**), the *Poc dhfr-ts* gene (**C**, **D**), and the *Poc mrp2* gene (**E**, **F**). EHH and haplotype bifurcation show selective sweeps spanning ~30, ~40, and ~30 kb, respectively, with lineage breakdown occurring first among the unselected allele haplotypes (blue) and then in the selected allele haplotypes (red) as the distance from the focal variant increases.

We calculated *n*S_L_ and Tajima’s D across the genomes to identify loci under directional and balancing selection, respectively. The *n*S_L_ statistic is considered robust to the currently unknown recombination rates across the genomes of *P. ovale* species^43^. Genetic markers of interest within 10 kb of the top 0.5% absolute normalized *n*S_L_ values that may be influenced by selective sweeps are listed in Tables 2, 3. Evidence of a selective sweep involving the putative bifunctional dihydrofolate reductase–thymidylate synthase (*dhfr-ts*) gene^41,42^ was found in both *P. ovale* species (Fig. 5). Examination of extended haplotype homozygosity (EHH) at the selected variants show a large selective sweep in *Pow* spanning roughly 40 kb as well as close proximity of the *dhfr-ts* gene to the focal variant (Fig. 6A, B). In *Poc*, the positioning of the *dhfr-ts* gene lies at the edge of a smaller sweep (Fig. 6C, D). However, another putative marker of drug resistance, multidrug resistance-associated protein 2 (*mrp2*), was found in close proximity to one of the highest absolute normalized *n*S_L_ values in *Poc* and lies near the center of a 40 kb sweep region on *Poc* chromosome 14 (Fig. 6E, F).

**Table 2:** Selection statistics and nearby genetic markers for loci with top normalized *n*S_L_ and positive Tajima’s *D* values among monoclonal isolates of *Poc*

Statistic	Value	Chr.	Location	Closest plausible genetic driver	Distance	Gene ID
***n*S**_**L**_	5.99	1	127,675	HECT-type E3 ubiquitin ligase UT, putative (*ut*)	1798	PocGH01_01012400
	−3.47	1	203,583	cysteine-rich secretory protein, putative (*crisp*)	-4763	PocGH01_01013600
	−3.61	3	541,949	cysteine repeat modular protein 2, putative (*crmp2*)	6695	PocGH01_03022100
	−3.40	5	66,310	early transcribed membrane protein, putative (*etramp*)	−790	PocGH01_05011300
	−3.38	5	107,531	GPI-anchored micronemal antigen, putative (*gama*)	−3142	PocGH01_05012100
	−3.71	5	774,030	bifunctional dihydrofolate reductase-thymidylate synthase, putative (*dhfr-ts*)	−9660	PocGH01_05028400
	−3.65	9	1,197,220	apical membrane antigen 1, putative (*ama1*)	605	PocGH01_09039800
	−3.55	12	703,579	merozoite surface protein 7-like protein, putative (*msp7*)	1983	PocGH01_12027700
	−4.03	12	2,760,110	male development protein, putative (*md1*)	0	PocGH01_12076000
	−4.01	14	1,607,839	AP2 domain transcription factor, putative (*ap2-g*)	−4111	PocGH01_14048300
	−5.25	14	1,899,110	ABC transporter C family member 2, putative (*mrp2*)	7715	PocGH01_14054800
**Tajima’s** ***D***	2.66	7	1,152,840	merozoite surface protein 1, putative (*msp1*)	0	PocGH01_07037900

Loci are described by chromosome (chr.), location on the chromosome in base pairs, and a statistical value in the top 0.5% across the genome. Nearby genetic markers were identified within 10,000 base pairs of these loci and are given alongside their gene ID and the distance of this marker to the reported locus (negative distance indicates upstream location). Source data are provided as a Source Data file.

**Table 3:** Selection statistics and nearby genetic markers for loci with top normalized *n*S_L_ and positive Tajima’s *D* values among monoclonal isolates of *Pow*

Statistic	Value	Chr.	Location	Closest plausible genetic driver	Distance	Gene ID
***n*S**_**L**_	−4.61	2	537994	dynein heavy chain, putative (*dhc*)	0	POWCR01_020017200
	−4.79	2	554712	transcription factor with AP2 domain(s) (*apiap2*)	7318	POWCR01_020017600
	−5.08	3	554677	dynein heavy chain, putative (*dhc*)	2069	POWCR01_030017700
	−4.99	4	603847	merozoite surface protein 5, putative (*msp5*)	−5083	POWCR01_040018700
	−4.89	5	846693	bifunctional dihydrofolate reductase-thymidylate synthase, putative (*dhfr-ts*)	−953	POWCR01_050023500
	−4.69	7	456511	cysteine repeat modular protein 1, putative (*crmp1*)	−6204	POWCR01_070013600
	−5.45	7	1080681	dynein light chain, putative (*dhc*)	8560	POWCR01_070029300
	−5.22	8	1135323	6-cysteine protein B9 (*6-cys*)	4768	POWCR01_080029500
	−4.39	9	792065	dynein heavy chain, putative (*dhc*)	0	POWCR01_090024400
	−4.93	12	525249	sporozoite protein essential for cell traversal, putative (*spect1*)	−5947	POWCR01_120016200
	−4.10	13	177064	*Plasmodium* interspersed repeat protein (*pir*)	−5114	POWCR01_130008000
	−4.10	13	177064	early transcribed membrane protein, putative (*etramp*)	6011	POWCR01_130008100
**Tajima’s D**	2.55	1	31910	conserved Plasmodium protein, unknown function	0	POWCR01_010005700
	2.58	1	128700	serine/threonine protein kinase, putative	0	POWCR01_070032200
	2.13	7	1184250	merozoite surface protein 1, putative (*msp1*)	0	POWCR01_080035700
	2.81	12	235390	stromal-processing peptidase, putative	0	POWCR01_130008300

Loci are described by chromosome (chr.), location on the chromosome in base pairs, and a statistical value in the top 0.5% of absolute values across the genome. Nearby genetic markers were identified within 10,000 base pairs of these loci and are given alongside their gene ID and the distance of this marker to the reported locus (negative distance indicates upstream location). Source data are provided as a Source Data file.

Top absolute *n*S_L_ hits were also found near *ap2* transcription factor genes that regulate apicomplexan life cycle transitions, including sexual differentiation into gametocytes (*ap2-g*), and genes involved in sex-specific development of gametes, such as those coding male development protein 1 (*md1*) and cysteine-rich secretory protein (*crisp*)^44–46^. In *Pow*, four top *n*S_L_ hits were found around genes encoding the dynein heavy and light chains, cytoskeleton components highly expressed in male gametes for motility and fusion with female gametes in the mosquito blood meal^47^. Top *n*S_L_ hits were also found near cysteine repeat modular proteins 2 and 1 (*crmp2*/*1)* in *Poc* and *Pow*, respectively, proteins which may be involved in targeting sporozoites to the salivary glands in the mosquito prior to transmission^48,49^.

Finally, genes encoding putative antigenic targets at the host-parasite interface, including merozoite surface protein 7 (*msp7*), merozoite surface protein 5 (*msp5*), early transcribed membrane protein (*etramp*), apical membrane antigen 1 (*ama1*), GPI-anchored micronemal antigen (*gama*), and 6-cysteine protein B9 (*6-cys*) may be under directional selection in both *P. ovale* species^50,51^. An orthologue of sporozoite protein essential for cell traversal 1 (*spect-1*), a protein necessary for liver cell invasion that has been investigated as a potential vaccine target^52^, was also among the top hits in *Pow*.

**Fig. 7:**
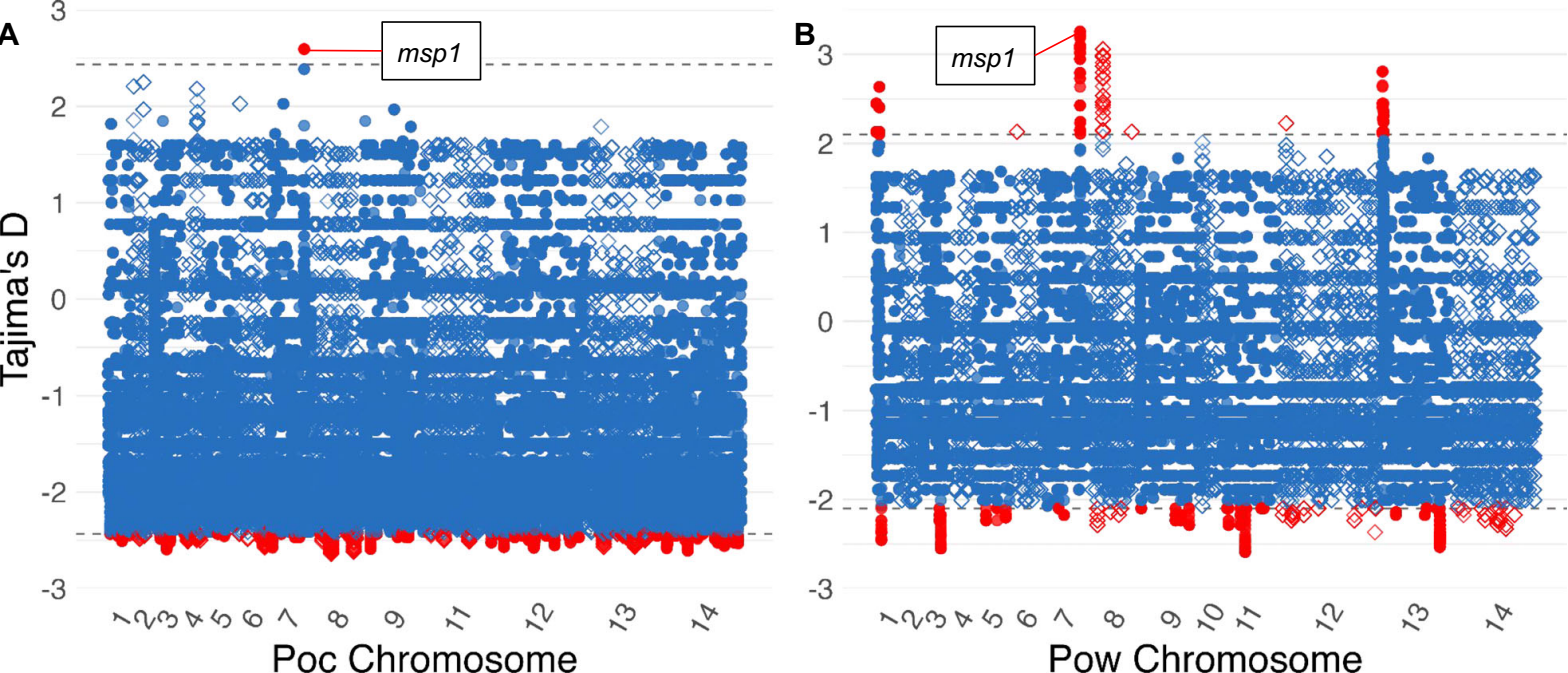
Tajima's D across the *Poc* and *Pow* genomes. Tajima’s D in 399,355 and 297,286 300 bp windows in genes across the *Poc* (**A**) and *Pow* (**B**) genomes among monoclonal isolates. Individual windows are depicted using alternating shapes between chromosomes for legibility. The dotted line and red color denote the top 0.5% of loci. For both species, the top locus with a positive Tajima’s D was located inside the merozoite surface protein 1 (*msp1*) gene. Source data are provided as a Source Data file.

Overall, Tajima’s D in both species exhibited a negative skew across the genome with an average value of −1.06 for *Poc* and −0.78 for *Pow*. This may suggest population expansion following a bottleneck or weak directional selection (Fig. 7). Among the loci with positive values in the top 0.5% of absolute Tajima’s D hits, the antigenic marker merozoite surface protein 1 (MSP1) was identified as a probable target of balancing or diversifying selection in both *P. ovale* species (Tables 2, 3).

## Discussion

We present a comprehensive population genomic study of both *P. ovale* species within sub-Saharan Africa. Our study comprises 21 *Poc* and 24 *Pow* isolates selected from 11 studies, including both febrile and asymptomatic cases. Genome-wide analysis reveals differences in nucleotide diversity between *P. ovale* species, but similarity in their low complexity of infection, geographic relatedness, and signatures of selection. Our analysis was performed using genomic enrichment methods specifically designed to enable robust coverage and analysis with the 2017 reference genomes of *P. ovale curtisi* (from Ghana) and *P. ovale wallikeri* (from Cameroon), which were the available references at the time of the study^28^.

Compared to the “classic” *P. ovale curtisi* species, we observed significantly lower nucleotide diversity across orthologous genes among geographically matched *P. ovale wallikeri* isolates (2.5 × 10^−4^ for *Poc* and 1.8 × 10^−4^ for *Pow*, respectively). Our estimate for *Poc* is concordant with the genome-wide diversity calculated among six Central African *Poc* isolates^15^ as well as that derived from RNA expression data among four parasite samples from Mali^53^. However, our *Pow* estimate was substantially lower than the genome-wide estimate reported by Higgins et al. (3.4 × 10^−4^), despite our inclusion of their samples alongside additional Central and East African isolates. Our lower estimate may reflect the exclusion of higher-diversity intergenic regions, though we also found lower genome-wide and intergenic SNP density in *Pow* compared to *Poc*. Relatively low nucleotide diversity in *Pow* may indicate reduced effective population size, increased inbreeding, or a population bottleneck in the time since *Poc* and *Pow* diverged between 1.3 and 20.3 million years ago^14,28^. More recent population growth in both species is also suggested by the predominantly negative distribution of Tajima’s D values across their protein-coding genes, a finding that can indicate population expansion following a bottleneck^54^. The high ratio of nonsynonymous-to-synonymous substitutions among these protein-coding genes (2.5 for *Pow*, 1.5 for *Poc*) is similar to that seen in *P. falciparum* and *P. vivax*^55,56^. This finding may represent diversifying selection on proteins across either *P. ovale* genome, enabling maintenance of nonsynonymous substitutions, or inflation of dN/dS ratios observed among *Plasmodium* parasites due to the impact of the malaria life cycle on allele frequencies^57^. Further analysis of subpopulations of each parasite species could help to elucidate the factors driving the observed difference in genomic diversity, such as by determining whether *Pow* isolates from Asia have similarly low nucleotide diversity or if this finding is specific to Africa.

The observed predominance of monoclonal isolates among both *P. ovale* species is consistent with low within-sample haplotypic diversity seen in previous investigations of African *P. ovale* isolates by genome-wide RNA sequencing and amplicon sequencing^53,58^. The low complexity of *P. ovale* infections may result from efficient clonal transmission^32^ and/or lower transmission overall, limiting vector uptake of multiple parasite clones from either the same or different infected individuals. This low complexity is expected to limit opportunities for genetic recombination within mosquito vectors, though multiple-clone infections were identified in Kinshasa, a region with overall higher malaria endemicity and transmission intensity^39^.

Genomic signatures of selection within both *P. ovale* species highlighted the importance of antimalarials, host-vector life cycle transitions, and human immunity as evolutionary pressures impacting parasite survival. *P. ovale* infections are frequently subclinical and go untreated^59^, but likely still face substantial drug exposure from widely used antimalarials prescribed for *P. falciparum*^22^. Additionally, malaria prophylaxis using sulfadoxine-pyrimethamine, such as intermittent preventive therapy in pregnancy (IPTp) and seasonal malaria chemoprevention for infants and schoolchildren (SMC), may be applying drug pressure on *P. ovale* parasite populations^1^. Selective sweeps in *dhfr-ts*, a gene implicated in resistance to pyrimethamine, have been documented in both *Poc* and *Pow*, and certain mutant alleles were found to confer pyrimethamine resistance when expressed in *E. coli*^24,25^. In our dataset, sweeps near the *dhfr-ts* genes were among the strongest signals of directional selection in both *P. ovale* species, especially in *Pow*, possibly representing drug pressure influencing parasite survival. We also found the putative pyrimethamine resistance *Pow dhfr-ts* haplotype Phe57Leu + Ser58Arg to be a major contributor to principal component 2 of *Pow* (representing the North-South axis); the resistant haplotype composed 36% (9/25) of our *Pow* haplotypes, with representation in Central and East Africa but no detection in West Africa. Another putative pyrimethamine resistance haplotype (Ala15Ser + Ser58Arg) in *Poc* was similarly detected in East and Central Africa, though not in West Africa^25^. Functional evaluation of different alleles to determine their capacity to confer drug resistance, as well as monitoring of these alleles across the parasite populations over time, will further clarify how interventions targeting *P. falciparum* may be simultaneously rendering *P. ovale* parasites harder to control.

Finally, strong signals of balancing or diversifying selection were observed in both species within their genes encoding merozoite surface protein 1 (MSP1), the orthologues of the predominant antigen on blood-stage *P. falciparum* parasites that has been shown to induce protective immunity in some studies^60^. Diversifying selection on MSP1 has been documented in *P. falciparum*^61^ as well as in focused analysis among African *P. ovale* infections imported to China^62^. Our dataset provides even stronger evidence for diversifying selection at this site, as the *msp1* gene showed the single highest Tajima’s D value across all genes in both *P. ovale* species. Such immune responses may also play a role in modulating relapse potential^63^.

This study has several limitations. While it represents the largest genome-wide examination of the genomic composition of both *P. ovale curtisi* and *P. ovale wallikeri* to date, the sample size for both species is nonetheless small and limits the power to detect clustering of isolates, infer population demography, and detect selection. The geographic coverage of the isolates employed differs between the 13 countries represented, and isolates from Northern or Southern Africa were not available. Whole-genome enrichment methods also differed among isolates; 21 isolates employed hybrid capture, 15 used selective whole-genome amplification, and nine relied on leukodepletion. The two former methods may have induced amplification bias, whereas leukodepletion does not amplify *P. ovale* DNA and, therefore, may reduce the power to identify rare variants in those isolates. Disparate average read depth between the three species (44.1, 95.7, and 147.5 for *Poc*, *Pow*, and *Pf*, respectively) may also have differentially impacted our ability to detect polyclonal infections, but the low complexity of infection found in both *P. ovale* species should be robust given the satisfactory sequencing depth overall. The source studies also differ in whether samples were derived from asymptomatic carriers (*n* = 11) or febrile patients (*n* = 34). Sample sizes were too small to analyze these populations separately. Finally, hybrid capture baits designed using the incomplete PowCR01 reference genome led to incomplete coverage of *Poc* chromosome 10, which was excluded from analyses. Unfortunately, newly assembled regions of *Pow* chromosome 10 were not available during the analysis. The hybrid capture approach also did not enable enrichment and analysis of loci in the mitochondrial and apicoplast genomes, which were excluded from analysis. We do not expect these exclusions to systematically bias the estimation of nucleotide diversity or complexity of infection, though it does prevent us from evaluating excluded loci (including *mdr1*, *msp3*, and *msp8*) for genomic signatures of selection. The availability of selective whole genome amplification protocols now provides a less expensive approach to targeted DNA enrichment for *P. ovale* spp. that does not rely on the specific design of hybrid capture baits^27^.

This study provides a comparative genomic analysis of the two *Plasmodium ovale* species sympatrically circulating in sub-Saharan Africa and presents new evidence of selective pressures on genes related to drug response, sexual differentiation, and immune evasion. Further population genomic studies of *Poc* and *Pow* should employ a larger selection of isolates from a greater geographic range, especially including Asia, and take advantage of new reference genome assemblies to build on these insights^15^. Functional investigation into the genes showing signatures of selection, including via orthologue replacement in closely related *Plasmodium* species^64,65^, is also an exciting new strategy in substantiating the biological relevance of key loci, with implications for transmission prevention, treatment strategies, and vaccine development for *P. ovale* spp. Finally, cataloging genome-wide diversity facilitates the design of targeted genotyping methods that can efficiently characterize the epidemiology of these understudied parasite species^66^. Combining these approaches to better evaluate *P. ovale* parasite relatedness, transmission, and relapse patterns can help to improve the impact of current malaria control strategies on all human-infecting malaria species^36,67,68^.

## Methods

### Ethics

This analysis was considered non-human subjects research by the University of North Carolina. The appropriate collection of samples, authorization for use in genomic studies, and IRBs involved are summarized in the original studies outlined in Supplemental Table 1.

### Sample selection

Clinical isolates in the form of dried blood spots or leukodepleted blood were drawn from six studies shown in Table 1, including studies involving both asymptomatic persons and febrile patients across four countries. Across these studies, participants were screened for the presence of *P. ovale* spp. infection by a real-time polymerase chain reaction (qPCR) assay targeting the *po18S* rRNA gene^22^. Among 282 isolates with a *po18S* Ct value under 40, a species-specific (*Poc* and *Pow*) 18S rRNA qPCR assay was employed to determine the ovale species present^69^. Candidates were selected from isolates with only one species detected or mixed infections in which one species predominated by ≥3 Ct (corresponding to approximately 8 times as much DNA). Samples were also screened for the presence of *P. falciparum* coinfection using a qPCR assay for the *pfldh* or *pf18S* rRNA gene^31,39^. Ultimately, samples from 25 individuals were selected for whole-genome sequencing based on higher-density *P. ovale* infection, lack of or lower-density *P. falciparum* coinfection, and balance of ovale species and geographic diversity across the sample set. Characteristics of these 25 samples, and an additional 20 samples from four previously published studies^15,28,36,37^, are shown in Supplemental Table 1.

### Library preparation and sequencing

DNA extracted from dried blood spots using a Chelex protocol^70^ was sheared to 300 bp using a LE220R-plus Covaris Sonicator (Covaris, Woburn, MA). Fragment size was checked with an Agilent TapeStation 4150 (Agilent, Santa Clara, CA), and DNA concentrations were tested using a Qubit Flex fluorometer (Thermo Fisher Scientific, Waltham, MA). Isolates were then prepared for sequencing using the KAPA Hyperprep kit (Kapa Biosystems, Woburn, MA). Four Tanzanian DNA isolates extracted from blood that had been leukocyte-depleted by CF11 filtration at the time of collection^35^ were directly incorporated into sequencing libraries. The remaining 21 isolates derived from dried blood spots were additionally processed using a custom-designed hybrid capture protocol to enrich for ovale DNA via thousands of RNA probes specifically designed to amplify *P. ovale* DNA without binding to human DNA (Twist Bioscience, San Francisco, CA, USA). Hybrid-capture probes were designed first for the *P. ovale wallikeri* genome (PowCR01), with unique probes added for the *P. ovale curtisi* genome (PocGH01) at any sites that differed by more than 10% of bases^28^. Since the reference genome for chromosome 10 is significantly smaller for *Poc* compared to *Pow* (roughly 1300 vs 470 kb, respectively), this bait design approach led to a lack of baits covering 63% of *Poc* chromosome 10. Chromosome 10 was therefore excluded from the analysis of *Poc* isolates. Captures were performed with four samples per capture. After preliminary sequencing on a Miseq Nano flow cell (Illumina, San Diego, CA, USA), libraries were sequenced on the Novaseq 6000 S Prime (Illumina, San Diego, CA, USA) sequencing system with 150 bp paired-end chemistry. Samples from Joste et al. and Higgins et al. included in our analysis were enriched using selective whole genome amplification (sWGA), employing sets of five to ten primers designed to preferentially amplify the PowCR01 and PocGH01 genomes over human background DNA^71^.

### Sequencing data alignment and variant calling

Data processing and analysis were performed in the bash environment using a Python-based *snakemake* v7.24.2 wrapper for pipeline construction, automation, and reproducibility^72^. Raw sequencing reads were trimmed of Illumina adapters using *Trimmomatic* v0.36^73^ before the quality of the reads was evaluated with *fastQC* v0.11.9^74^. A “dual” reference genome was then produced for each *P. ovale* species by concatenating the reference genome of that ovale species (*P. ovale curtisi: PocGH01; P. ovale wallikeri: PowCR01*) to the *P. falciparum* strain *Pf3D7* reference genome^28,75^. Paired reads from each isolate were aligned to their species’ corresponding dual reference genome using *bwa-mem2* v2.2.1^76^ before each alignment was sorted and given read group information using *picard* v2.26.11^77^ and deduplicated via *GATK* v4.4.0.0^78^. *Samtools* v1.17 was then used to select for reads that aligned to the *P. ovale* portion of the dual reference genome rather than that of *P. falciparum*, thus discarding reads from contaminating *P. falciparum* DNA present in some isolates^79^. Resulting alignments of read that preferentially mapped to *P. ovale* were soft-clipped to reference genome edges and cleaned of unmapped reads using *GATK*, after which mapping proportion and coverage of the ovale reference genome were calculated by *samtools* and *bedtools* v2.30^80^.

Variant calling from aligned reads across each ovale species genome was also performed using the *GATK* best practices pipeline^78^. In the resulting callset, variants were masked via *vcftools*^81^ if they fell outside of the 14 chromosomes of the reference genome or were within the following expanded gene families (which were masked to reduce sequence error in repetitive genomic elements): *Plasmodium* interspersed repeat protein (*pir*), surfin-related subtelomeric protein 1 (*stp1*), early transcribed membrane protein (*etramp*), tryptophan-rich antigen (*tra*), *Plasmodium* exported protein (*phist)*, reticulocyte binding protein (*rbp*), ookinete surface protein (*osp*), 6-cysteine protein, KELT protein, and pm-fam-a protein^37^. Tandem repeats across each species’ genome were identified and masked using Tandem Repeat Finder v4.09.1^82^. *GATK* hard filtering was then used to remove variants with poor quality metrics using the following filter thresholds: quality by depth <3, Fisher strand bias >50, strand odds ratio >3, mapping quality >50, mapping quality rank sum <−2.5, read position rank sum <−3. Call sets were limited to biallelic single nucleotide polymorphisms (SNPs) that were present in at least 80% of individuals. SNP density across the entire genome and within specific functional regions of each genome were calculated using custom scripts (see Data availability). *SNPeff* v4.3 was used to annotate individual variants and determine the ratio of nonsynonymous-to-synonymous mutations^28,83^.

### Selection of *P. falciparum* comparison dataset

Co-endemic *P. falciparum* samples were drawn from the Pf6 dataset^38^. Of 20,705 total *P. falciparum* isolates from around the globe, 2077 came from the same or nearby geographic locations as the source studies of *P. ovale* isolates described above. Thirty-two *P. falciparum* samples with over 85% base callability were randomly selected in order to have at least one co-endemic *P. falciparum* isolate for each *P. ovale curtisi* or *ovale wallikeri* isolate (Supplemental Table 2). Variant call sets for these *P. falciparum* samples were limited to the falciparum core genome^75^, quality filtered by Variant Quality Score Recalibration^78^, and restricted to sites present in at least 80% of individuals.

### Complexity of infection (COI) calculation

Variant call sets were limited to variants with a minor allele frequency greater than or equal to 5%. The McCOILR R package was employed to run THEREALMcCOIL on each sample set using 1000 Markov chain Monte Carlo iterations, 100 burn-in iterations, a maximum COI of 25, a minimum number of sites for a sample to be included of 10, and a minimum number of samples for a site to be included of 10 (analysis was run on 21 and 24 *P. ovale curtisi* and *ovale wallikeri* isolates, respectively)^84^. This yielded 1000 estimates of the complexity of infection, or the number of unique parasite clones found in each sample, and median COI per isolate was reported. COI distributions among *P. falciparum* isolates were calculated in the same manner. COI distributions among the three species were compared using a Kruskal–Wallis test, with Dunn’s multiple comparisons test employed for specific pairwise comparisons^85^.

To investigate the distribution of heterozygous SNPs in samples that were determined to be polyclonal, the variant set was filtered to sites with >10% minor allele frequency across the whole population and a within-sample minor allele frequency >50% for at least one sample. Then, for each polyclonal sample, the remaining sites were filtered to those with a within-sample minor allele frequency greater than or equal to 5%.

### Principal components analysis

After limiting to monoclonal samples (20 *Poc*, 23 *Pow*), variant call sets were filtered to remove sites with a minor allele frequency of less than 5% using *vcftools* v0.1.15^81^. Variants were processed using PLINK v1.90b6.21, first by pruning variants within windows of 50 variants between which the R^2^ value exceeded 0.3 (windows were shifted by steps of five variants for each pruning step)^86^. Then, principal components analysis was performed in PLINK, including extraction of the weights by variant. Data visualizations were performed in R using the *ggplot2* v3.4.4 package^87^. ADMIXTURE v1.3.0 was employed to test for significant clustering among samples within each species^88^.

### Nucleotide diversity calculation

For both ovale species and *P. falciparum*, the OrthoMCL database was searched for orthologous gene sets in which there was only one copy of each ortholog per genome^89^. Sets were removed from the total pool of orthologous genes if they were partially or totally masked by the multigene family or extrachromosomal masks within an individual species, or if any orthologs did not have ≥5x coverage of the gene at least every 10 bases in at least 60% of isolates in each species. Among the remaining ortholog sets with sufficient coverage among all species, nucleotide diversity (**π**) was calculated across the monoclonal samples of each species for each ortholog in the set using *vcftools* v0.1.15^81^. The *P. falciparum* isolate set was composed of 19 monoclonal isolates selected to ensure one geographically matched *Pf* sample for each *P. ovale* sample (Supplemental Table 2). Variant call sets in this analysis did not employ a minor allele frequency filter. Nucleotide diversity was compared between species among the remaining sets of orthologs using repeated measures ANOVA; pairwise comparisons were examined with two-tailed Tukey’s multiple comparisons test. To mitigate potential bias introduced by differing geographic coverage between the two ovale species (and by differences with the *P. falciparum* genome), nucleotide diversity was also calculated among a set of orthologue sets (identified among one-to-one *Poc*-*Pow* orthologues with strong coverage as detailed above) in geographically matched *Poc* and *Pow* samples (*n* = 11 each, Supplemental Table 3) and compared using Wilcoxon’s matched-pair signed rank test^90^.

### Identification of signatures of selection

Variant call sets were limited to monoclonal isolates and sites which had no missingness in any samples. After employing the default minor allele frequency filter of >5%, *selscan* v1.2.0 was used to calculate *n*S_L_ (a metric of directional selection) for all remaining variants in each species and the *norm* function used to normalize values in allele frequency bins^91^. *n*S_L_ was chosen as it is more robust to the currently unknown recombination rates across either *P. ovale* genome than metrics like iHS and because it can evaluate selection within a single population of organisms. Loci with an *n*S_L_ in the most extreme 0.5% were investigated in PlasmoDB for proximity to genes of interest within 10 kb^89^. Extended haplotype homozygosity (EHH) was calculated at select sites using *rehh*^92^. Tajima’s D (a metric of possible directional and balancing selection) was calculated in 300 base pair sliding windows shifted by 10 bp using all non-missing variants across all known genes in each genome using *vcf-kit* v0.2.9^93,94^. Variants with positive Tajima’s D values in the top 0.5% of absolute values were investigated in PlasmoDB.

### Reporting summary

Further information on research design is available in the Nature Portfolio Reporting Summary linked to this article.

## Supplementary information

Supplementary Information

Peer Review File

Reporting summary

## Source data

Source data

## Data Availability

All new sequence data are available at NCBI SRA: BioProject ID PRJNA1092086. Public data used include the contents of the following projects/studies: European Nucleotide Archive Study accession number PRJEB51041, Run accession numbers: ERR10738334, ERR10738339, ERR10738341, and ERR10738346. SRA Study accession number PRJEB13344 https://www.ncbi.nlm.nih.gov/bioproject/PRJEB13344], Run accession numbers: ERR1739852, and ERR1739853. SRA Study accession number PRJEB12679, Run accession numbers: ERR1428159, ERR1254542, and ERR1254543. SRA Study accession number PRJNA1015456, Run accession numbers: SRR26037552, SRR26037551, SRR26037550, SRR26037549, SRR26037548, SRR26037546, SRR26037545, SRR26037544, SRR26037543, SRR26037542, and SRR26037541. Additional Run accession numbers: ERR404145. ERR404154. ERR377533. ERR404191. ERR404207. ERR1045266. ERR1045267. ERR676479. ERR1106575. ERR1106579. ERR1106586. ERR1106587. ERR1106590. ERR449901. ERR449903. ERR405238. ERR405244. ERR666939. ERR562889. ERR636018 [https://www.ncbi.nlm.nih.gov/sra/?term=ERR562889]. ERR912913 [https://www.ncbi.nlm.nih.gov/sra/?term=ERR636018]. ERR1514567. ERR1045287. ERR1172616. ERR1172593. ERR1172615. ERR1172608. ERR059405. ERR045598. ERR666937. ERR580480. ERR701763 Source data are provided with this paper.

## Code availability

All code for processing and analysis of samples is available at: Carey-Ewend, K., Population genomics of Plasmodium ovale species in sub-Saharan Africa, https://github.com/bailey-lab/Po_popgen_snakemake/tree/main/final, 10.5281/zenodo.14026786^95^.

## References

[1] World Health Organization. W*orld Malaria Report 2023* (World Health Organization, 2023).

[2] Lover, A. A., Baird, J. K., Gosling, R. & Price, R. N. Malaria elimination: time to target all species. *Am. J. Trop. Med. Hyg.* **99**, 17–23 (2018). 29761762 10.4269/ajtmh.17-0869 PMC6035869

[3] Betson, M., Clifford, S. & Stanton, M. Emergence of nonfalciparum *Plasmodium* infection despite regular artemisinin combination therapy in an 18-month longitudinal study of Ugandan children and their mothers. *J. Infect. Dis.* **217**, 1099–1109 (2018). 29325068 10.1093/infdis/jix686 PMC5939692

[4] Yman, V. et al. Persistent transmission of *Plasmodium malariae* and *Plasmodium ovale* species in an area of declining *Plasmodium falciparum* transmission in eastern Tanzania. *PLoS Negl. Trop. Dis.* **13**, e0007414 (2019). 31136585 10.1371/journal.pntd.0007414 PMC6555537

[5] Akala, H. M. et al. *Plasmodium* interspecies interactions during a period of increasing prevalence of Plasmodium ovale in symptomatic individuals seeking treatment: an observational study. *Lancet Microbe* **2**, e141–e150 (2021). 10.1016/S2666-5247(21)00009-4 PMC7613221 35544189

[6] Amato, R. et al. Genetic markers associated with dihydroartemisinin–piperaquine failure in *Plasmodium falciparum* malaria in Cambodia: a genotype–phenotype association study. *Lancet Infect. Dis.* **17**, 164–173 (2017). 27818095 10.1016/S1473-3099(16)30409-1 PMC5564489

[7] Bailey, J. A. et al. Microarray analyses reveal strain-specific antibody responses to *Plasmodium falciparum* apical membrane antigen 1 variants following natural infection and vaccination. *Sci. Rep.* **10**, 3952 (2020). 32127565 10.1038/s41598-020-60551-z PMC7054363

[8] Amambua-Ngwa, A. et al. Major subpopulations of *Plasmodium falciparum* in sub-Saharan Africa. *Science* **365**, 813–816 (2019). 31439796 10.1126/science.aav5427

[9] Volkman, S. K. et al. A genome-wide map of diversity in *Plasmodium falciparum*. *Nat. Genet.* **39**, 113–119 (2007). 17159979 10.1038/ng1930

[10] Ghansah, A. et al. Monitoring parasite diversity for malaria elimination in sub-Saharan Africa. *Science* **345**, 1297–1298 (2014). 25214619 10.1126/science.1259423 PMC4541720

[11] Collins, W. E. & Jeffery, G. M. *Plasmodium ovale*: parasite and disease. *Clin. Microbiol. Rev.* **18**, 570–581 (2005). 16020691 10.1128/CMR.18.3.570-581.2005 PMC1195966

[12] Mueller, I., Zimmerman, P. A. & Reeder, J. C. *Plasmodium malariae* and *Plasmodium ovale*-the ‘bashful’malaria parasites. *Trends Parasitol.* **23**, 278–283 (2007). 17459775 10.1016/j.pt.2007.04.009 PMC3728836

[13] Calderaro, A. et al. Genetic polymorphisms influence *Plasmodium ovale* PCR detection accuracy. *J. Clin. Microbiol.* **45**, 1624–1627 (2007). 17360843 10.1128/JCM.02316-06 PMC1865880

[14] Sutherland, C. J. et al. Two nonrecombining sympatric forms of the human malaria parasite *Plasmodium ovale* occur globally. *J. Infect. Dis.* **201**, 1544–1550 (2010). 20380562 10.1086/652240

[15] Higgins, M. et al. New reference genomes to distinguish the sympatric malaria parasites, *Plasmodium ovale**curtisi* and *Plasmodium ovale* wallikeri. *Sci. Rep.* **14**, 3843 (2024). 38360879 10.1038/s41598-024-54382-5 PMC10869833

[16] Šlapeta, J., Sutherland, C. J. & Fuehrer, H.-P. Calling them names: variants of *Plasmodium ovale*. *Trends Parasitol*. 10.1016/j.pt.2023.12.010 (2023) 10.1016/j.pt.2023.12.010 38160179

[17] Snounou, G., Sharp, P. M. & Culleton, R. Appropriate naming of the two *Plasmodium ovale* species. *Trends Parasitol.* **40**, 207–208 (2024). 38272740 10.1016/j.pt.2024.01.004

[18] Snounou, G., Sharp, P. M. & Culleton, R. The two parasite species formerly known as *Plasmodium ovale*. *Trends Parasitol.* **40**, 21–27 (2024). 38040603 10.1016/j.pt.2023.11.004

[19] Oguike, M. C. et al. *Plasmodium ovale**curtisi* and *Plasmodium ovale**wallikeri* circulate simultaneously in African communities. *Int. J. Parasitol*. **41**, 677–683 (2011). 10.1016/j.ijpara.2011.01.004 PMC3084460 21315074

[20] Fuehrer, H.-P. et al. *Plasmodium ovale* in Bangladesh: genetic diversity and the first known evidence of the sympatric distribution of *Plasmodium ovale**curtisi* and *Plasmodium ovale**wallikeri* in southern Asia. *Int. J. Parasitol.* **42**, 693–699 (2012). 22633951 10.1016/j.ijpara.2012.04.015

[21] Fuehrer, H.-P., Campino, S. & Sutherland, C. J. The primate malaria parasites *Plasmodium malariae*, *Plasmodium brasilianum* and *Plasmodium ovale* spp.: genomic insights into distribution, dispersal and host transitions. *Malaria J*. **21**10.1186/s12936-022-04151-4 (2022). 10.1186/s12936-022-04151-4 PMC9066925 35505317

[22] Sendor, R. et al. Similar prevalence of *Plasmodium falciparum* and non–*P. falciparum* malaria infections among schoolchildren, Tanzania. *Emerg. Infect. Dis. J.* **29**, 1143 (2023). 10.3201/eid2906.221016 PMC10202886 37209670

[23] Lei, Y. et al. Low genetic diversity and strong immunogenicity within the apical membrane antigen-1 of *Plasmodium ovale* spp. imported from africa to china. *Acta Trop.* **210**, 105591 (2020). 32562621 10.1016/j.actatropica.2020.105591 PMC7456792

[24] Chen, J. et al. Disparate selection of mutations in the dihydrofolate reductase gene (dhfr) of *Plasmodium ovale**curtisi* and *P. o. wallikeri* in Africa. *PLoS Negl. Trop. Dis.* **16**, e0010977 (2022). 36469541 10.1371/journal.pntd.0010977 PMC9754596

[25] Joste, V. et al. *Plasmodium ovale* spp dhfr mutations associated with reduced susceptibility to pyrimethamine in sub-Saharan Africa: a retrospective genetic epidemiology and functional study. *Lancet Microbe* **5**, 669–678 (2024). 38761813 10.1016/S2666-5247(24)00054-5

[26] Bright, A. T. et al. Whole genome sequencing analysis of *Plasmodium vivax* using whole genome capture. *BMC Genomics* **13**, 262 (2012). 22721170 10.1186/1471-2164-13-262 PMC3410760

[27] Joste, V., Guillochon, E., Clain, J., Coppée, R. & Houzé, S. Development and optimization of a selective whole-genome amplification to study *Plasmodium ovale* Spp. *Microbiol. Spectr*. **10**, e0072622 (2022). 10.1128/spectrum.00726-22 PMC9602584 36098524

[28] Rutledge, G. G. et al. *Plasmodium malariae* and *P. ovale* genomes provide insights into malaria parasite evolution. *Nature* **542**, 101–104 (2017). 10.1038/nature21038 PMC5326575 28117441

[29] Feleke, S. M. et al. *Plasmodium falciparum* is evolving to escape malaria rapid diagnostic tests in Ethiopia. *Nat. Microbiol.* **6**, 1289–1299 (2021). 34580442 10.1038/s41564-021-00962-4 PMC8478644

[30] Parr, J. B. et al. Analysis of false-negative rapid diagnostic tests for symptomatic malaria in the Democratic Republic of the Congo. *Sci. Rep.* **11**, 6495 (2021). 33753817 10.1038/s41598-021-85913-z PMC7985209

[31] Mwandagalirwa, M. K. et al. Individual and household characteristics of persons with *Plasmodium falciparum* malaria in sites with varying endemicities in Kinshasa Province, Democratic Republic of the Congo. *Malar. J.* **16**, 456 (2017). 29121931 10.1186/s12936-017-2110-7 PMC5680818

[32] Tarimo, B. B. et al. Seasonality and transmissibility of *Plasmodium ovale* in Bagamoyo District, Tanzania. *Parasit. Vectors* **15**, 56 (2022). 35164867 10.1186/s13071-022-05181-2 PMC8842944

[33] Rogier, E. et al. *Plasmodium falciparum* pfhrp2 and pfhrp3 gene deletions among patients enrolled at 100 health facilities throughout Tanzania: February to July 2021. *Sci Rep* **14**, 8158 (2024). 10.1038/s41598-024-58455-3 PMC11001933 38589477

[34] Ali, I. M. et al. Arboviruses as an unappreciated cause of non-malarial acute febrile illness in the Dschang Health District of western Cameroon. *PLoS Negl. Trop. Dis.* **16**, e0010790 (2022). 36223421 10.1371/journal.pntd.0010790 PMC9591055

[35] Venkatesan, M. et al. Using CF11 cellulose columns to inexpensively and effectively remove human DNA from *Plasmodium falciparum*-infected whole blood samples. *Malar. J.* **11**, 41 (2012). 22321373 10.1186/1475-2875-11-41 PMC3295709

[36] Joste, V. et al. Genetic profiling of *Plasmodium ovale* wallikeri relapses with microsatellite markers and whole-genome sequencing. *J. Infect. Dis.* **228**, 1089–1098 (2023). 37329228 10.1093/infdis/jiad216

[37] Ansari, H. R. et al. Genome-scale comparison of expanded gene families in *Plasmodium ovale* wallikeri and *Plasmodium ovale**curtisi* with *Plasmodium malariae* and with other *Plasmodium* species. *Int. J. Parasitol.* **46**, 685–696 (2016). 27392654 10.1016/j.ijpara.2016.05.009

[38] MalariaGEN et al. An open dataset of *Plasmodium falciparum* genome variation in 7,000 worldwide samples. *Wellcome Open Res.* **6**, 42 (2021). 33824913 10.12688/wellcomeopenres.16168.1 PMC8008441

[39] Sendor, R. et al. Epidemiology of *Plasmodium malariae* and *Plasmodium ovale* spp. in Kinshasa Province, Democratic Republic of Congo. *Nat. Commun.* **14**, 6618 (2023). 37857597 10.1038/s41467-023-42190-w PMC10587068

[40] Bright, A. T. et al. A high resolution case study of a patient with recurrent *Plasmodium vivax* infections shows that relapses were caused by meiotic siblings. *PLoS Negl. Trop. Dis.* **8**, e2882 (2014). 24901334 10.1371/journal.pntd.0002882 PMC4046966

[41] Sirawaraporn, W., Sathitkul, T., Sirawaraporn, R., Yuthavong, Y. & Santi, D. V. Antifolate-resistant mutants of Plasmodium falciparum dihydrofolate reductase. *Proc. Natl Acad. Sci. USA* **94**, 1124–1129 (1997). 9037017 10.1073/pnas.94.4.1124 PMC19755

[42] Hastings, M. D. et al. Dihydrofolate reductase mutations in *Plasmodium vivax* from Indonesia and therapeutic response to sulfadoxine plus pyrimethamine. *J. Infect. Dis.* **189**, 744–750 (2004). 14767830 10.1086/381397

[43] Ferrer-Admetlla, A., Liang, M., Korneliussen, T. & Nielsen, R. On detecting incomplete soft or hard selective sweeps using haplotype structure. *Mol. Biol. Evol.* **31**, 1275–1291 (2014). 24554778 10.1093/molbev/msu077 PMC3995338

[44] Gomes, A. R. et al. A transcriptional switch controls sex determination in *Plasmodium falciparum*. *Nature* **612**, 528–533 (2022). 36477538 10.1038/s41586-022-05509-z PMC9750867

[45] Russell, A. J. C. et al. Regulators of male and female sexual development are critical for the transmission of a malaria parasite. *Cell Host Microbe* **31**, 305–319.e10 (2023). 36634679 10.1016/j.chom.2022.12.011 PMC7616090

[46] Tadesse, F. G. et al. Gametocyte sex ratio: the key to understanding *Plasmodium falciparum* transmission? *Trends Parasitol.* **35**, 226–238 (2019). 30594415 10.1016/j.pt.2018.12.001 PMC6396025

[47] Khan, S. M. et al. Proteome analysis of separated male and female gametocytes reveals novel sex-specific *Plasmodium* biology. *Cell* **121**, 675–687 (2005). 15935755 10.1016/j.cell.2005.03.027

[48] Douradinha, B. et al. *Plasmodium* cysteine repeat modular proteins 3 and 4 are essential for malaria parasite transmission from the mosquito to the host. *Malar. J.* **10**, 71 (2011). 21453484 10.1186/1475-2875-10-71 PMC3083381

[49] Thompson, J. et al. Plasmodium cysteine repeat modular proteins 1-4: complex proteins with roles throughout the malaria parasite life cycle. *Cell. Microbiol.* **9**, 1466–1480 (2007). 17253978 10.1111/j.1462-5822.2006.00885.x

[50] Lyons, F. M. T., Gabriela, M., Tham, W.-H. & Dietrich, M. H. *Plasmodium* 6-cysteine proteins: functional diversity, transmission-blocking antibodies and structural scaffolds. *Front. Cell. Infect. Microbiol.* **12**, 945924 (2022). 35899047 10.3389/fcimb.2022.945924 PMC9309271

[51] Arumugam, T. U. et al. Discovery of GAMA, a *Plasmodium falciparum* merozoite micronemal protein, as a novel blood-stage vaccine candidate antigen. *Infect. Immun.* **79**, 4523–4532 (2011). 21896773 10.1128/IAI.05412-11 PMC3257921

[52] Patarroyo, M. E., Alba, M. P. & Curtidor, H. Biological and structural characteristics of the binding peptides from the sporozoite proteins essential for cell traversal (SPECT)-1 and -2. *Peptides* **32**, 154–160 (2011). 20933029 10.1016/j.peptides.2010.09.026

[53] Tebben, K. et al. Malian children infected with *Plasmodium ovale* and *Plasmodium falciparum* display very similar gene expression profiles. *PLoS Negl. Trop. Dis.* **17**, e0010802 (2023). 36696438 10.1371/journal.pntd.0010802 PMC9901758

[54] Tajima, F. Statistical method for testing the neutral mutation hypothesis by DNA polymorphism. *Genetics* **123**, 585–595 (1989). 2513255 10.1093/genetics/123.3.585 PMC1203831

[55] Singh, G. P. & Sharma, A. South-East Asian strains of *Plasmodium falciparum* display higher ratio of non-synonymous to synonymous polymorphisms compared to African strains. *F1000Res.* **5**, 1964 (2016). 27853513 10.12688/f1000research.9372.1 PMC5089136

[56] Feng, X. et al. Single-nucleotide polymorphisms and genome diversity in *Plasmodium vivax*. *Proc. Natl Acad. Sci. USA* **100**, 8502–8507 (2003). 12799466 10.1073/pnas.1232502100 PMC166258

[57] Chang, H.-H. et al. Malaria life cycle intensifies both natural selection and random genetic drift. *Proc. Natl Acad. Sci. USA* **110**, 20129–20134 (2013). 24259712 10.1073/pnas.1319857110 PMC3864301

[58] Saralamba, N. et al. Genetic dissociation of three antigenic genes in *Plasmodium ovale curtisi* and *Plasmodium ovale wallikeri*. *PLoS ONE* **14**, e0217795 (2019). 31170213 10.1371/journal.pone.0217795 PMC6553752

[59] Faye, F. B. K. et al. Diagnostic criteria and risk factors for *Plasmodium ovale* malaria. *J. Infect. Dis.* **186**, 690–695 (2002). 12195357 10.1086/342395

[60] Thomson-Luque, R., Stabler, T. C., Fürle, K., Silva, J. C. & Daubenberger, C. *Plasmodium falciparum* merozoite surface protein 1 as asexual blood stage malaria vaccine candidate. *Expert Rev. Vaccines* **23**, 160–173 (2024). 38100310 10.1080/14760584.2023.2295430

[61] Escalante, A. A., Lal, A. A. & Ayala, F. J. Genetic polymorphism and natural selection in the malaria parasite *Plasmodium falciparum*. *Genetics* **149**, 189–202 (1998). 9584096 10.1093/genetics/149.1.189 PMC1460124

[62] Chu, R. et al. Limited genetic diversity of N-terminal of merozoite surface protein-1 (MSP-1) in *Plasmodium ovale**curtisi* and *P. ovale**wallikeri* imported from Africa to China. *Parasit. Vectors* **11**, 596 (2018). 30446012 10.1186/s13071-018-3174-0 PMC6240192

[63] Joyner, C. J. et al. Humoral immunity prevents clinical malaria during Plasmodium relapses without eliminating gametocytes. *PLoS Pathog.* **15**, e1007974 (2019). 31536608 10.1371/journal.ppat.1007974 PMC6752766

[64] Thiam, L. G., Mangou, K., Ba, A., Mbengue, A. & Bei, A. K. Leveraging genome editing to functionally evaluate *Plasmodium* diversity. *Trends Parasitol.* **38**, 558–571 (2022). 35469746 10.1016/j.pt.2022.03.005

[65] Mohring, F. et al. Cation ATPase (ATP4) orthologue replacement in the malaria parasite *Plasmodium knowlesi* reveals species-specific responses to ATP4-targeting drugs. *MBio* **13**, e0117822 (2022). 36190127 10.1128/mbio.01178-22 PMC9600963

[66] Siegel, S. V. et al. Lineage-informative microhaplotypes for recurrence classification and spatio-temporal surveillance of *Plasmodium vivax* malaria parasites. *Nat. Commun.* **15**, 6757 (2024). 39117628 10.1038/s41467-024-51015-3 PMC11310204

[67] Liu, P. et al. Increasing proportions of relapsing parasite species among imported malaria in China’s Guangxi Province from Western and Central Africa. *Travel Med. Infect. Dis.* **43**, 102130 (2021). 34166802 10.1016/j.tmaid.2021.102130 PMC8429216

[68] Wångdahl, A. et al. Relapse of *Plasmodium vivax* and *Plasmodium ovale* malaria with and without primaquine treatment in a nonendemic area. *Clin. Infect. Dis.* **74**, 1199–1207 (2022). 34216464 10.1093/cid/ciab610 PMC8994585

[69] Potlapalli, V. R. et al. Real-time PCR detection of mixed *Plasmodium ovale**curtisi* and *wallikeri* infections in human and mosquito hosts. *PLoS Negl. Trop. Dis.* **17**, e0011274 (2023). 38064489 10.1371/journal.pntd.0011274 PMC10732364

[70] Mitchell, C. L. et al. Under the radar: epidemiology of *Plasmodium ovale* in the Democratic Republic of the Congo. *J. Infect. Dis.* **223**, 1005–1014 (2021). 32766832 10.1093/infdis/jiaa478 PMC8006425

[71] Clarke, E. L. et al. swga: a primer design toolkit for selective whole genome amplification. *Bioinformatics* **33**, 2071–2077 (2017). 28334194 10.1093/bioinformatics/btx118 PMC5870857

[72] Mölder, F. et al. Sustainable data analysis with Snakemake. *F1000Res.* **10**, 33 (2021). 34035898 10.12688/f1000research.29032.1 PMC8114187

[73] Bolger, A. M., Lohse, M. & Usadel, B. Trimmomatic: a flexible trimmer for Illumina sequence data. *Bioinformatics* **30**, 2114–2120 (2014). 24695404 10.1093/bioinformatics/btu170 PMC4103590

[74] Wingett, S. W. & Andrews, S. FastQ Screen: a tool for multi-genome mapping and quality control. *F1000Res.* **7**, 1338 (2018). 30254741 10.12688/f1000research.15931.1 PMC6124377

[75] Miles, A. et al. Indels, structural variation, and recombination drive genomic diversity in *Plasmodium falciparum*. *Genome Res.* **26**, 1288–1299 (2016). 27531718 10.1101/gr.203711.115 PMC5052046

[76] Vasimuddin, M., Misra, S., Li, H. & Aluru, S. Efficient architecture-aware acceleration of BWA-MEM for multicore systems. In *2019 IEEE International Parallel and Distributed Processing Symposium (IPDPS)* 314–324 (IEEE, 2019).

[77] Institute, B. ‘Picard Toolkit’, Broad institute, GitHub repository (2021).

[78] Van der Auwera, G. A. & O’Connor, B. D. *Genomics in the Cloud: Using Docker, GATK, and WDL in Terra* (O’Reilly Media, Inc., 2020).

[79] Li, H. et al. The sequence alignment/map format and SAMtools. *Bioinformatics* **25**, 2078–2079 (2009). 19505943 10.1093/bioinformatics/btp352 PMC2723002

[80] Quinlan, A. R. & Hall, I. M. BEDTools: a flexible suite of utilities for comparing genomic features. *Bioinformatics* **26**, 841–842 (2010). 20110278 10.1093/bioinformatics/btq033 PMC2832824

[81] Danecek, P. et al. The variant call format and VCFtools. *Bioinformatics* **27**, 2156–2158 (2011). 21653522 10.1093/bioinformatics/btr330 PMC3137218

[82] Benson, G. Tandem repeats finder: a program to analyze DNA sequences. *Nucleic Acids Res.* **27**, 573–580 (1999). 9862982 10.1093/nar/27.2.573 PMC148217

[83] Cingolani, P. et al. A program for annotating and predicting the effects of single nucleotide polymorphisms, SnpEff: SNPs in the genome of Drosophila melanogaster strain w1118; iso-2; iso-3. *Fly* **6**, 80–92 (2012). 22728672 10.4161/fly.19695 PMC3679285

[84] Chang, H.-H. et al. THE REAL McCOIL: a method for the concurrent estimation of the complexity of infection and SNP allele frequency for malaria parasites. *PLoS Comput. Biol.* **13**, e1005348 (2017). 28125584 10.1371/journal.pcbi.1005348 PMC5300274

[85] Dunn, O. J. Multiple comparisons among means. *J. Am. Stat. Assoc.* **56**, 52–64 (1961).

[86] Purcell, S. et al. PLINK: a tool set for whole-genome association and population-based linkage analyses. *Am. J. Hum. Genet.* **81**, 559–575 (2007). 17701901 10.1086/519795 PMC1950838

[87] Wickham, H. *ggplot2: Elegant Graphics for Data Analysis* (Springer Science & Business Media, 2009).

[88] Alexander, D. H., Novembre, J. & Lange, K. Fast model-based estimation of ancestry in unrelated individuals. *Genome Res.* **19**, 1655–1664 (2009). 19648217 10.1101/gr.094052.109 PMC2752134

[89] Amos, B. et al. VEuPathDB: the eukaryotic pathogen, vector and host bioinformatics resource center. *Nucleic Acids Res.* **50**, D898–D911 (2022). 34718728 10.1093/nar/gkab929 PMC8728164

[90] Conover, W. J. *Practical Nonparametric Statistics* 3rd edn (John Wiley & Sons, 1999).

[91] Szpiech, Z. A. & Hernandez, R. D. selscan: an efficient multithreaded program to perform EHH-based scans for positive selection. *Mol. Biol. Evol.* **31**, 2824–2827 (2014). 10.1093/molbev/msu211 PMC4166924 25015648

[92] Gautier, M. & Vitalis, R. rehh: an R package to detect footprints of selection in genome-wide SNP data from haplotype structure. *Bioinformatics* **28**, 1176–1177 (2012). 22402612 10.1093/bioinformatics/bts115

[93] Parobek, C. M. et al. Selective sweep suggests transcriptional regulation may underlie *Plasmodium vivax* resilience to malaria control measures in Cambodia. *Proc. Natl Acad. Sci. USA* **113**, E8096–E8105 (2016). 27911780 10.1073/pnas.1608828113 PMC5167194

[94] Cook, D. E. & Andersen, E. C. VCF-kit: assorted utilities for the variant call format. *Bioinformatics* **33**, 1581–1582 (2017). 28093408 10.1093/bioinformatics/btx011 PMC5423453

[95] Carey-Ewend, K. Population genomics of *Plasmodium ovale* species in sub-Saharan Africa. *Zenodo*10.5281/zenodo.14026786 (2024). 10.1038/s41467-024-54667-3 PMC11603351 39604397

