# Supplementary material for: Population genomics of Plasmodium ovale species in sub-Saharan Africa
Source: Nat Commun. 2024 Nov 27;15:10297. doi: 10.1038/s41467-024-54667-3 (PMC11603351; doi:10.1038/s41467-024-54667-3)
Supplement: Supplementary file 1 — Supplementary Information [file 41467_2024_54667_MOESM1_ESM.pdf]

Population Genomics of *Plasmodium ovale* Species in Sub-Saharan Africa

Supplemental Figures and Tables

Sample ID	*po18S* Ct value	*P. ovale* Species	*P. ovale* species Ct value (*Poc*, *Pow*)	Technique	Country of Origin	Study Site	*Pf* Coinfection	1x Cov. (%)	5x Cov. (%)	10x Cov. (%)	Average Mean Depth
DSG272	34.2	ovale curtisi	37.0, 50.0	HC	Cameroon	Dschang	negative	96.9	95.3	94.4	67.4
SRR26037543poc7	N/A	ovale curtisi	N/A	sWGA	Cameroon	Imported to UK	negative	98.8	84.1	51.0	9.9
366152	29.4	ovale curtisi	34.8, -	HC	DRC	Bas-Uele	negative	97.1	95.3	94.3	131.0
364184	31.5	ovale curtisi	38.5, -	HC	DRC	Bas-Uele	negative	95.8	93.2	89.3	23.0
3073204	29.5	ovale curtisi	30.9, -	HC	DRC	Kinshasa	negative	96.1	94.3	93.1	76.2
1031006	32.3	ovale curtisi	35.3, -	HC	DRC	Kinshasa	positive	96.8	95.4	94.6	72.2
1013003	32.5	ovale curtisi	34.5, -	HC	DRC	Kinshasa	positive	96.2	94.5	93.2	62.5
1011201	34.0	ovale curtisi	35.1, -	HC	DRC	Kinshasa	positive	95.6	92.1	87.9	26.9
1012505	34.7	ovale curtisi	36.2, -	HC	DRC	Kinshasa	positive	95.5	92.5	89.3	30.8

2714 A	33.2	ovale curtisi	34.6, -	HC	Ethiopia	Amhara	negative	95.3	91.7	86.8	20.9
3116 A	31.8	ovale curtisi	32.5, -	HC	Ethiopia	Amhara	negative	95.9	94.0	92.4	34.4
ERR1739852pocgh01	N/A	ovale curtisi	N/A	LDB	Ghana	Upper East	positive	99.6	99.5	99.3	46.2
SRR26037545poc5	N/A	ovale curtisi	N/A	sWGA	Nigeria	Imported to UK	negative	99.4	94.8	73.5	13.6
SRR26037544poc6	N/A	ovale curtisi	N/A	sWGA	Nigeria	Imported to UK	negative	99.6	96.6	83.0	17.3
SRR26037542poc8	N/A	ovale curtisi	N/A	sWGA	Nigeria	Imported to UK	negative	99.5	98.5	92.3	22.2
ERR1428159povcu2	N/A	ovale curtisi	N/A	LDB	Nigeria	Imported to China	negative	99.6	99.3	98.6	62.2
SRR26037541poc9	N/A	ovale curtisi	N/A	sWGA	Sierra Leone	Imported to UK	negative	99.5	96.6	82.0	15.7
SRR26037546poc4	N/A	ovale curtisi	N/A	sWGA	South Sudan	Imported to UK	negative	99.6	99.4	99.2	85.8
1610 D0	32.2	ovale curtisi	34.8, -	LDB	Tanzania	Pwani	positive	99.4	94.9	71.2	14.6
426	34.0	ovale curtisi	40.0, -	LDB	Tanzania	Pwani	positive	99.5	99.2	98.6	82.3
475	35.6	ovale curtisi	41.1, -	LDB	Tanzania	Pwani	positive	97.6	75.8	41.3	10.4
1507081819_r2a	N/A	ovale wallikeri	N/A	sWGA	Cameroon	Imported to France	negative	95.8	78.0	53.9	16.3

1802062292 _r4a	N/A	ovale wallikeri	N/A	sWGA	Cameroon	Imported to France	negative	98.5	97.7	95.0	63.9
ERR173985 3powcr01	N/A	ovale wallikeri	N/A	LDB	Cameroon	Southwes t	positive	98.4	96.1	75.7	12.2
SRR260375 48pow16	N/A	ovale wallikeri	N/A	sWGA	Congo	Imported to UK	negative	98.5	96.5	86.0	19.0
111038	33.0	ovale wallikeri	-, 34.7	HC	DRC	Kinshasa	negative	97.0	94.4	88.5	425.6
113135	30.6	ovale wallikeri	44.4, 35.2	HC	DRC	Kinshasa	negative	98.5	98.2	97.9	547.8
353176	32.7	ovale wallikeri	45.4, 35.7	HC	DRC	Bas-Uele	negative	97.8	97.4	97.2	86.1
242012	32.9	ovale wallikeri	47.8, 40.7	HC	DRC	Sud-Kivu	negative	97.9	97.4	97.2	110.4
2090A	33.5	ovale wallikeri	-, 40.8	HC	Ethiopia	Amhara	negative	98.0	97.5	97.3	54.4
2562 DA	34.0	ovale wallikeri	-, 39.2	HC	Ethiopia	Amhara	negative	98.1	97.6	97.3	65.8
2589 DA	32.1	ovale wallikeri	-, 38.1	HC	Ethiopia	Amhara	negative	97.6	97.4	97.2	60.5
2680 A	31.5	ovale wallikeri	-, 38.3	HC	Ethiopia	Amhara	negative	98.2	97.8	97.6	142.6
2928 A	34.1	ovale wallikeri	-, 41.1	HC	Ethiopia	Amhara	negative	98.0	97.5	97.2	50.0
ERR125454 3povwa2	N/A	ovale wallikeri	N/A	LDB	Gabon	Imported to China	negative	98.6	98.5	98.3	64.9

ERR1254542povwa1	N/A	ovale wallikeri	N/A	LDB	Gabon	Imported to China	negative	98.6	98.5	98.4	82.7
1503082061_r1a	N/A	ovale wallikeri	N/A	sWGA	Ivory Coast	Imported to France	negative	93.3	67.6	39.6	10.9
SRR26037551pow13	N/A	ovale wallikeri	N/A	sWGA	Kenya	Imported to UK	negative	98.5	94.9	82.2	20.7
SRR26037549pow15	N/A	ovale wallikeri	N/A	sWGA	Nigeria	Imported to UK	negative	98.6	98.3	97.8	71.6
1701100582_r3a	N/A	ovale wallikeri	N/A	sWGA	Senegal	Imported to France	negative	98.2	96.8	93.0	58.5
SRR26037550pow14	N/A	ovale wallikeri	N/A	sWGA	South Sudan	Imported to UK	negative	98.4	91.6	67.9	12.7
SOMO_ISA_041	30.0	ovale wallikeri	42.5, 38.4	HC	Tanzania	Songwe	negative	97.8	97.4	97.2	81.3
SOTU_TUN_019	29.6	ovale wallikeri	41.1, 37.2	HC	Tanzania	Songwe	negative	97.9	97.5	97.4	128.7
123	32.2	ovale wallikeri	-, 43.8	LDB	Tanzania	Pwani	positive	98.5	98.1	97.3	99.5
SRR26037552pow12	N/A	ovale wallikeri	N/A	sWGA	Tanzania	Imported to UK	negative	97.2	80.3	51.9	11.7

Supplemental Table 1. Metadata of 21 *P. ovale curtisi* and 24 *P. ovale wallikeri* clinical isolates. Technique refers to the method used to enrich *P. ovale* genomic DNA and/or remove human DNA. For Ct values, "-" indicates no amplification. *Pf* co-infection status was determined by real-time polymerase chain reaction. For 20 samples incorporated from other studies, *po18S* Ct values were not available. HC = hybrid capture; LDB = leukodepleted blood sample; sWGA = selective whole-genome amplification; DRC = Democratic Republic of the Congo; *Pf* = *P. falciparum;* _x cov. (%) = percent of species core genome covered by ≥_ reads (excluding *Poc* chromosome 10).

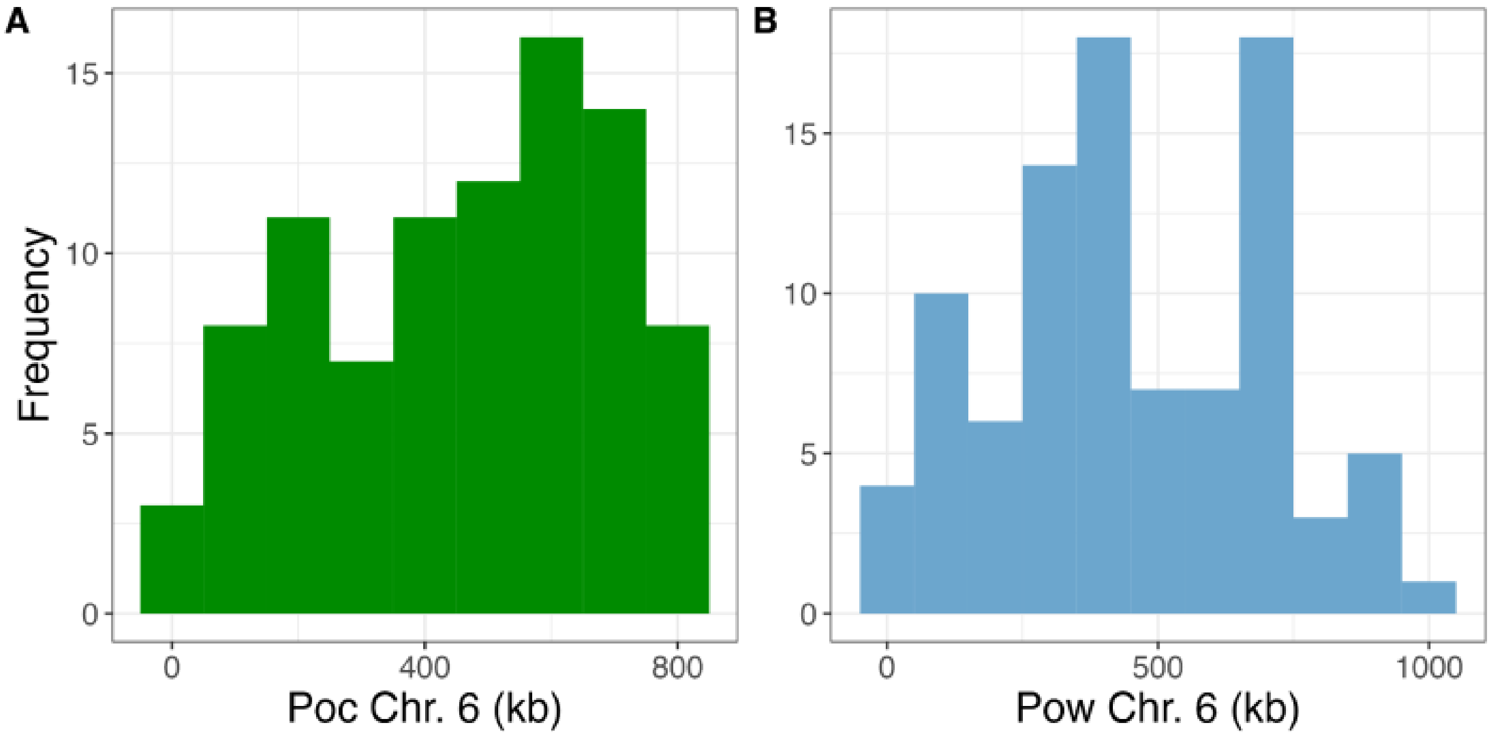

Supplemental Figure 1. Heterozygous calls across chromosome (chr.) 6 in one polyclonal *Poc* isolate (A) and one polyclonal *Pow* isolate (B). X-axis shows bins along the length of each chromosome in kilobases (kb); y-axis shows counts of heterozygous base calls in each isolate after filtering by minor allele frequency within the sample and across the corresponding population. Findings in chromosome 6 were representative of the remaining 13 chromosomes in both samples. Distribution of heterozygous calls across the full chromosome indicates that the two clones present in each isolate are not closely-related meiotic siblings.

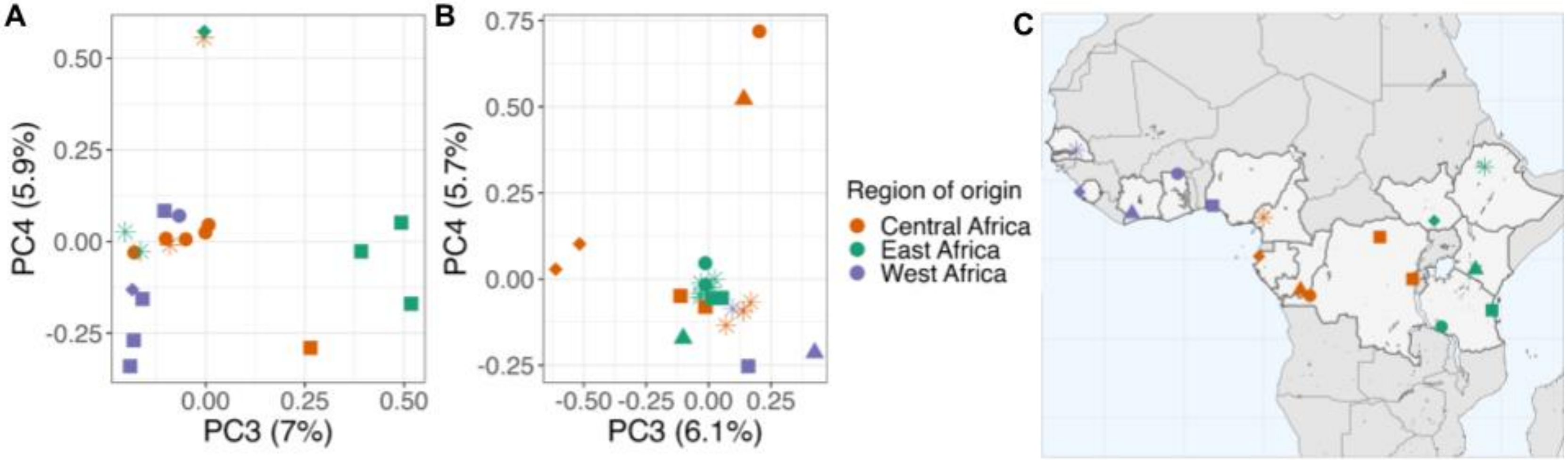

Supplemental Figure 2. Principal component analysis showing the second two principal components among (A) 20 monoclonal *Poc* isolates and (B) 23 monoclonal *Pow* isolates using 4,116 and 3,189 biallelic SNPs, respectively. Samples colored by region of country of origin; in the map, parasites from travelers are assigned to capital city (C). Source data are provided as a Source Data file.

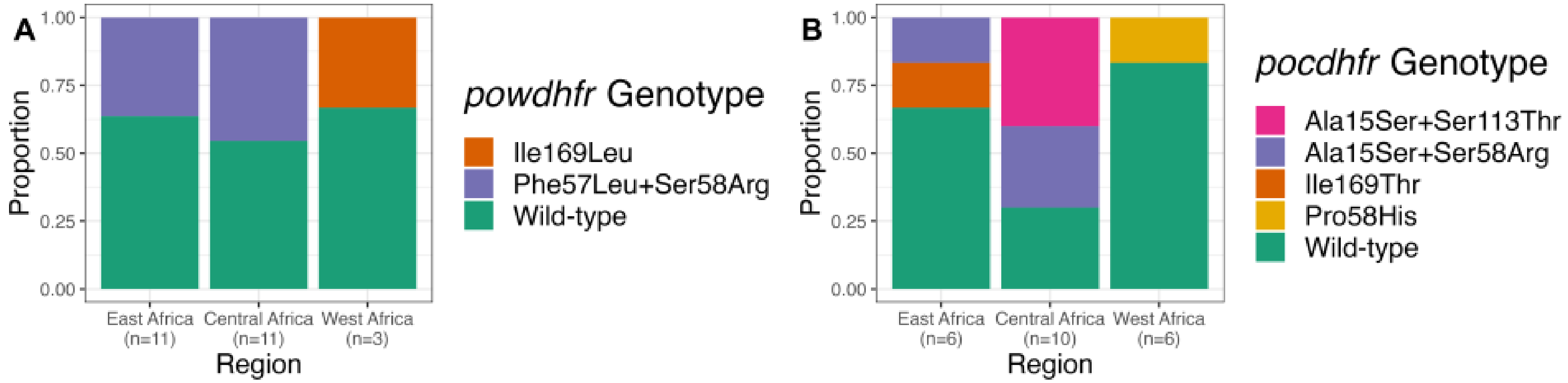

Supplemental Figure 3. *Pow* (A) and *Poc* (B) *dhfr-ts* genotypes by region. In *Pow,* SNPs in this gene were among the top 0.5% of contributors to principal component 2. One polyclonal Central African isolate of each species were estimated to contain two clones and were heterozygous for the wild-type and Phe57Leu+Ser58Arg haplotypes (in *Pow*) and the Ala15Ser+58Arg and Ala15Ser+Ser113Thr haplotypes (in *Poc*). The two clones of each isolate are plotted as separate observations in these graphs. Source data are provided as a Source Data file.

Pf Sample ID	Country	Region	% Callable	Corresponding *Po* Country	Corresponding *Po* Region
QG0007-C	DRC	Kinshasa	90.5	DRC	Kinshasa
QG0015-C	DRC	Kinshasa	90.4	DRC	Kinshasa
QG0140-C	DRC	Kinshasa	90.5	DRC	Kinshasa
QG0189-C	DRC	Kinshasa	90.0	DRC	Kinshasa
QG0270-C	DRC	Kinshasa	88.3	DRC	Kinshasa
QS0109-C	DRC	Kinshasa	90.3	DRC	Bas-Uele
QS0110-C	DRC	Kinshasa	90.0	DRC	Bas-Uele
PE0538-C	Tanzania	Kagera	90.4	DRC	Sud-Kivu
QS0154-C	Ethiopia	Amhara	88.6	Ethiopia	Amhara
QS0159-C	Ethiopia	Amhara	89.7	Ethiopia	Amhara
QS0168-C	Ethiopia	Amhara	87.4	Ethiopia	Amhara
QS0169-C	Ethiopia	Amhara	87.8	Ethiopia	Amhara
QS0170-C	Ethiopia	Amhara	88.8	Ethiopia	Amhara
PE0129-C	Tanzania	Morogoro	90.6	Tanzania	Songwe
PE0130-C	Tanzania	Morogoro	90.0	Tanzania	Songwe
PE0109-C	Tanzania	Tanga	90.1	Tanzania	Pwani
PE0113-C	Tanzania	Tanga	89.7	Tanzania	Dar es Salaam
QQ0111-C	Ivory Coast	Abidjan	90.3	Ivory Coast	Abidjan
QV0030-C	Senegal	Dakar	89.7	Senegal	Dakar
QP0254-C	Cameroon	Sud-Ouest	90.0	Cameroon	Dschang
QP0193-C	Cameroon	Sud-Ouest	90.1	Cameroon	Dschang
QG0360-C	DRC	Kinshasa	89.1	Congo	Brazzaville
QS0133-C	Ethiopia	Oromia	90.3	South Sudan	Central Equitoria
QJ0172-C	Nigeria	Lagos	90.0	Nigeria	Lagos
QJ0173-C	Nigeria	Lagos	90.7	Nigeria	Lagos
QJ0015-C	Nigeria	Lagos	90.5	Nigeria	Lagos
QJ0164-C	Nigeria	Lagos	88.2	Nigeria	Lagos
PA0234-C	Guinea	Forestière	91.0	Sierra Leone	Western Area
PF0309-C	Ghana	Upper East	90.0	Ghana	Upper East
QP0249-C	Gabon	Estuaire	90.6	Gabon	Estuaire
QP0048-C	Gabon	Estuaire	91.1	Gabon	Estuaire
PC0295-C	Kenya	Kisumu	90.3	Kenya	Kisumu

Supplemental Table 2. Selection of variant callsets for 32 *P. falciparum* (*Pf*) isolates from the Pf6 dataset to match the geographic distribution of *P. ovale* isolates. % callable refers to percent of SNPs that passed quality filtering by variant quality score recalibration against a validated set of *Pf* SNPs. If *P. ovale* isolates were drawn from a region or country from which there were no available *Pf* samples in the Pf6 dataset, the nearest country and region were selected.

Poc Sample ID	*Pow* Sample ID	Country
DSG272	1802062292_r4a	Cameroon
3073204	111038	DRC
364184	353176	DRC
1610 D0	123	Tanzania
426	SOMO_ISA_041	Tanzania
475	SOTU_TUN_019	Tanzania
2714 A	2680 A	Ethiopia
3116 A	2562 DA	Ethiopia
SRR26037543poc7	1507081819_r2a	Cameroon
SRR26037542poc8	SRR26037549pow15	Nigeria
SRR26037546poc4	SRR26037550pow14	South Sudan

Supplemental Table 3. Selection of geographically-matched *P. ovale curtisi* and *P. ovale wallikeri* isolates.
