# Supplementary material for: Population genomics of Plasmodium ovale species in sub-Saharan Africa
Source: Nat Commun. 2024 Nov 27;15:10297. doi: 10.1038/s41467-024-54667-3 (PMC11603351; doi:10.1038/s41467-024-54667-3)
Supplement: Supplementary file 3 — Reporting summary [file 41467_2024_54667_MOESM3_ESM.pdf]

nature portfolio

Corresponding author(s): Kelly Carey-Ewend

Last updated by author(s): Nov 15, 2024

Reporting Summary

Nature Portfolio wishes to improve the reproducibility of the work that we publish. This form provides structure for consistency and transparency in reporting. For further information on Nature Portfolio policies, see our Editorial Policies and the Editorial Policy Checklist.

Statistics

For all statistical analyses, confirm that the following items are present in the figure legend, table legend, main text, or Methods section.

n/a	Confirmed	
☐	☒	The exact sample size (*n*) for each experimental group/condition, given as a discrete number and unit of measurement
☐	☒	A statement on whether measurements were taken from distinct samples or whether the same sample was measured repeatedly
☐	☒	The statistical test(s) used AND whether they are one- or two-sided<br>*Only common tests should be described solely by name; describe more complex techniques in the Methods section.*
☐	☒	A description of all covariates tested
☐	☒	A description of any assumptions or corrections, such as tests of normality and adjustment for multiple comparisons
☐	☒	A full description of the statistical parameters including central tendency (e.g. means) or other basic estimates (e.g. regression coefficient) AND variation (e.g. standard deviation) or associated estimates of uncertainty (e.g. confidence intervals)
☐	☒	For null hypothesis testing, the test statistic (e.g. *F*, *t*, *r*) with confidence intervals, effect sizes, degrees of freedom and *P* value noted<br>*Give P values as exact values whenever suitable.*
☒	☐	For Bayesian analysis, information on the choice of priors and Markov chain Monte Carlo settings
☒	☐	For hierarchical and complex designs, identification of the appropriate level for tests and full reporting of outcomes
☒	☐	Estimates of effect sizes (e.g. Cohen's *d*, Pearson's *r*), indicating how they were calculated

Our web collection on statistics for biologists contains articles on many of the points above.

Software and code

Policy information about availability of computer code

Data collection	All code for processing and analysis of samples is available at https://github.com/bailey-lab/Po_popgen_snakemake/tree/main/final and https://doi.org/10.5281/zenodo.14026786. Additional links to publicly available data sets are described in Data Availability.
Data analysis	All code for processing and analysis of samples is available at https://github.com/bailey-lab/Po_popgen_snakemake/tree/main/final and https://doi.org/10.5281/zenodo.14026786. Package and software versions are specified in Methods and Supplemental Methods

For manuscripts utilizing custom algorithms or software that are central to the research but not yet described in published literature, software must be made available to editors and reviewers. We strongly encourage code deposition in a community repository (e.g. GitHub). See the Nature Portfolio guidelines for submitting code & software for further information.

Data

Policy information about availability of data

All manuscripts must include a data availability statement. This statement should provide the following information, where applicable:

- Accession codes, unique identifiers, or web links for publicly available datasets
- A description of any restrictions on data availability
- For clinical datasets or third party data, please ensure that the statement adheres to our policy

All new sequence data are available at NCBI SRA (BioProject ID: PRJNA1092086). Public data used include European Nucleotide Archive Study Accession Number: PRJEB51041 (Run Accession Numbers: ERR10738334, ERR10738339, ERR10738341, ERR10738346), SRA Study Accession Number: PRJEB13344 (Run Accession

Numbers: ERR1739852, ERR1739853), SRA Study Accession Number: PRJEB12679 (Run Accession Numbers: ERR1428159, ERR1254542, ERR1254543), SRA Study Accession Numbers: PRJNA1015456 (Run Accession Numbers: SRR26037552, SRR26037551, SRR26037550, SRR26037549, SRR26037548, SRR26037546, SRR26037545, SRR26037544,, SRR26037543, SRR26037542, SRR26037541), and additional Run Accession Numbers: ERR404145, ERR404154, ERR377533, ERR404191, ERR404207, ERR1045266, ERR1045267, ERR676479, ERR1106575, ERR1106579, ERR1106586, ERR1106587, ERR1106590, ERR449901, ERR449903, ERR405238, ERR405244, ERR666939, ERR562889, ERR636018, ERR912913, ERR1514567, ERR1045287, ERR1172616, ERR1172593, ERR1172615, ERR1172608, ERR059405, ERR045598, ERR666937, ERR580480, ERR701763.

Research involving human participants, their data, or biological material

Policy information about studies with human participants or human data. See also policy information about sex, gender (identity/presentation), and sexual orientation and race, ethnicity and racism.

Reporting on sex and gender	Demographic metadata on research subjects from whom parasite samples were collected were not incorporated into analyses in this manuscript, which was focused on genomic characteristics of the parasites themselves.
Reporting on race, ethnicity, or other socially relevant groupings	Demographic metadata on research subjects from whom parasite samples were collected were not incorporated into analyses in this manuscript, which was focused on genomic characteristics of the parasites themselves.
Population characteristics	Demographic metadata on research subjects from whom parasite samples were collected were not incorporated into analyses in this manuscript, which was focused on genomic characteristics of the parasites themselves.
Recruitment	This analysis was considered non-human subjects research by the University of North Carolina. The appropriate collection of samples and IRBs involved are summarized in the original studies outlined in Supplemental Table 1.
Ethics oversight	This analysis was considered non-human subjects research by the University of North Carolina. The appropriate collection of samples and IRBs involved are summarized in the original studies outlined in Supplemental Table 1.

Note that full information on the approval of the study protocol must also be provided in the manuscript.

Field-specific reporting

Please select the one below that is the best fit for your research. If you are not sure, read the appropriate sections before making your selection.

☐ Life sciences ☐ Behavioural & social sciences ☒ Ecological, evolutionary & environmental sciences

For a reference copy of the document with all sections, see nature.com/documents/nr-reporting-summary-flat.pdf

Ecological, evolutionary & environmental sciences study design

All studies must disclose on these points even when the disclosure is negative.

Study description	We performed whole-genome sequencing of 29 P. ovale curtisi and P. ovale wallikeri parasite isolates collected from seven studies of malaria conducted across sub-Saharan Africa in order to describe population genomic characteristics of the two parasite populations in endemic settings.
Research sample	P. ovale curtisi and P. ovale wallikeri DNA was identified in dried blood spots and leukodepleted blood samples using highly-sensitive polymerase chain reaction. After isolation from blood samples, parasite DNA then underwent whole-genome sequencing and was analyzed at a population level.
Sampling strategy	Sample size calculations were not performed. The final sample set was selected from available isolates based on suitability for sequencing, concentration of parasite DNA, geographic range, species diversity, and availability of reagents for hybrid capture enrichment. Differences in sample size between the two P. ovale species studied largely represent differences in availability and quality of parasite isolates.
Data collection	Data was collected from seven malaria surveillance studies described in Table 1. Blood samples were collected from participants following informed consent and were stored at appropriate temperatures (see "Field-collected Samples") prior to shipment to UNC for additional screening, processing, and analysis.
Timing and spatial scale	Sampling of various malaria-endemic populations in Africa occurred from 2013-2022 across the seven studies described in Table 1. The spatial scope was selected to represent parasites across central, west, and east Africa. As secondary use of samples from surveillance studies of malaria, the spatial and time scale of sample collection is also influenced by the individuals purposes of those studies (see references for Table 1).
Data exclusions	No relevant data exclusions. Details of inclusion of specific samples into specific analyses are described in Methods and Supplemental Methods.
Reproducibility	This observational study into the genomics of two neglected malaria parasite species did not have an experimental component and could not be replicated within the scope of the study.

Randomization	Randomization was not relevant because this was not an experimental study.
Blinding	Blinding was not relevant because this was not an experimental study.

Did the study involve field work? ☒ Yes ☐ No

Field work, collection and transport

Field conditions	The various studies from which the isolates in this study were collected were conducted across Africa from 2013-2022 (though certain isolates from external studies did not include date of collection), representing a range of climate, geographic, and social/ demographic conditions that are not directly relevant to this analysis of the genomic characteristics of parasite populations.
Location	Studies were conducted across the Democratic Republic of the Congo, Tanzania, Ethiopia, Cameroon, Senegal, and the Ivory Coast (see Table 1).
Access & import/export	Samples were exported from their collection sites in Africa in accordance with the policies of local governments, institutional collaborators in country, and the UNC IRB. Details of this export can be found in references for each of the original studies (see Table 1).
Disturbance	The secondary use of parasite DNA from isolates already collected as part of malaria surveillance studies did not impose any significant disturbance on local populations.

Reporting for specific materials, systems and methods

We require information from authors about some types of materials, experimental systems and methods used in many studies. Here, indicate whether each material, system or method listed is relevant to your study. If you are not sure if a list item applies to your research, read the appropriate section before selecting a response.

Materials & experimental systems

n/a	Involved in the study	
☒	☐	Antibodies
☒	☐	Eukaryotic cell lines
☒	☐	Palaeontology and archaeology
☐	☒	Animals and other organisms
☒	☐	Clinical data
☒	☐	Dual use research of concern
☒	☐	Plants

Methods

n/a	Involved in the study	
☒	☐	ChIP-seq
☒	☐	Flow cytometry
☒	☐	MRI-based neuroimaging

Animals and other research organisms

Policy information about studies involving animals; ARRIVE guidelines recommended for reporting animal research, and Sex and Gender in Research

Laboratory animals	No laboratory animals employed in this study.
Wild animals	No wild animals were employed in this study.
Reporting on sex	Findings of this study refer to Plasmodium parasites which are not sexually-differentiated during most stages of infection.
Field-collected samples	This study largely comprised secondary use of field samples collected from multiple preexisting studies (see Table 1). Dried blood spots were stored in refrigerated conditions down to 4 degrees Celsius while leukodepleted blood samples were stored at -80 degrees Celsius until the point of parasite DNA isolation by chelex extraction. DNA samples were subsequently stored at -20 degrees Celsius prior to sequencing. Samples were collected from 2013-2022 (depending on study) and were typically shipped from their collection site in Africa to UNC for processing within 1-3 years of collection.
Ethics oversight	This analysis was considered non-human subjects research by the University of North Carolina. The appropriate collection of samples and IRBs involved are summarized in the original studies outlined in Supplemental Table 1.

Note that full information on the approval of the study protocol must also be provided in the manuscript.

Plants

Seed stocks	No plants were involved in this study.
Novel plant genotypes	No plants were involved in this study.
Authentication	No plants were involved in this study.
